# The Species and Origin of Shark Fins in Taiwan’s Fishing Ports, Markets, and Customs Detention: A DNA Barcoding Analysis

**DOI:** 10.1371/journal.pone.0147290

**Published:** 2016-01-22

**Authors:** Po-Shun Chuang, Tzu-Chiao Hung, Hung-An Chang, Chien-Kang Huang, Jen-Chieh Shiao

**Affiliations:** Institute of Oceanography, National Taiwan University, Taipei, Taiwan; Aristotle University of Thessaloniki, GREECE

## Abstract

The increasing consumption of shark products, along with the shark’s fishing vulnerabilities, has led to the decrease in certain shark populations. In this study we used a DNA barcoding method to identify the species of shark landings at fishing ports, shark fin products in retail stores, and shark fins detained by Taiwan customs. In total we identified 23, 24, and 14 species from 231 fishing landings, 316 fin products, and 113 detained shark fins, respectively. All the three sample sources were dominated by *Prionace glauca*, which accounted for more than 30% of the collected samples. Over 60% of the species identified in the fin products also appeared in the port landings, suggesting the domestic-dominance of shark fin products in Taiwan. However, international trade also contributes a certain proportion of the fin product markets, as four species identified from the shark fin products are not found in Taiwan’s waters, and some domestic-available species were also found in the customs-detained sample. In addition to the species identification, we also found geographical differentiation in the *cox1* gene of the common thresher sharks (*Alopias vulpinus*), the pelagic thresher shark (*A*. *pelagicus*), the smooth hammerhead shark (*Sphyrna zygaena*), and the scalloped hammerhead shark (S. *lewini*). This result might allow fishing authorities to more effectively trace the origins as well as enforce the management and conservation of these sharks.

## Introduction

Shark, once regarded mainly as a by-catch product with low value [1–3], have recently become a main conservation concern [4–6]. To satisfy its growing demand, Asia imports roughly 10,000–20,000 tons of shark fins per year for the purpose of consumption [7]. The huge annual consumption in China, which accounts for over 80% of the world’s shark trade [8], might play a major role in the overexploitation of shark resources. As a consequence, shark population decreases and collapses have been reported world-wide by several studies [9–11].

Characterized by a life history of slow growth, late maturity, and low fecundity, the shark is extremely vulnerable to overexploitation and has low population resilience to overfishing [11–13]. Because sharks are often at the top of marine food webs and play keystone roles in many ecosystems, the conservation of sharks is ecologically important [14–16]. Sustaining shark resources is also economically essential as shark fins are one of the most valuable types of seafood in Asia [17–19]. However, the current practice shark fisheries use negates the goals of species conservation and population restoration. The shark finning practice wastes shark resources and hastens the collapse of shark populations. Although shark finning has been banned in many countries [3, 20], illegal shark fishing seems to continue [11, 20, 21]. To reinforce shark conservations, the Convention on the International Trade in Endangered Species of Wild Fauna and Flora (CITES) has recently added several shark species to Appendix II for international trade regulations, and regional fisheries management organizations (RFMOs) have enforced the anti-catch regulations of several cosmopolitan shark species. Nevertheless, these actions’ regulatory effects have been minimal, partly due to the difficulty in identifying a species and its origin.

Despite being rich in fishery resources and the 4^th^ largest shark-catching country in the world [12], Taiwan lacks information on the composition of its shark landings and shark products. Liu et al. (2013) have recently reported the species composition of shark flesh in Taiwan’s markets [19]. However, their study sampled shark meat products instead of processed shark fins, which are more common in domestic and international trade. To add to Taiwan’s knowledge base on this subject, we collected shark samples from fishing port landings and from marketed shark fin products in Taiwan. Since 2013, we have also been involved in examining the species of shark fins detained, as a result of not being declared for import, by Taiwan’s customs. Using the DNA barcoding method, we surveyed the species composition of the shark landings, the fin products, and the detained fins. In addition, we examined the *cox1* gene sequences of several shark species regulated by RFMOs to investigate whether ocean-specific characteristics could distinguish the shark landings of different ocean basins.

## Materials and Methods

### Sample collection

Sharks were usually caught as bycatch by Taiwanese longliners targeting on tunas. As a consequence, in the period of 2012 to 2014 we collected 231 fresh shark tissues from the three fishing ports where tuna-longliners majorly land (TungKang, XinGang, and NanFangAo). In the fishing ports of XinGang and NanFangAo, whole sharks were landed without finning. While for the landing in TungKang fishing port, the sharks were frozen with their fins removed and tied on the carcasses, which was allowed from 2012 to 2013 as a transition period after the implementation of the shark finning rule in Taiwan. All the samples were collected from fishermen belonging to the local fishermen’s associations (TungKang, XinGang, and Su-Ao, respectively), which are all belonging to the National Fishermen's Association, Taiwan, ROC. For all the samples from fishing ports, a small piece of the caudal fins, including the surrounding skin and muscle, were cut out from the sharks and stored at -20°C.

In the period of 2012 to 2014, we purchased 316 fin products from retail stores in four cities (Taipei, Taichung, Changhua, and Kaohsiung) in Taiwan. The purchased samples included various sizes (0.14 to 50.74 g) and shapes (e.g., right triangle, equilateral triangle, and irregular shapes). Detail information for each individual sample collected in this study is provided in S1 Table. The dry fin samples were stored in darkness until the analysis. In addition, we collected 113 samples from shark fins detained by Kaohsiung customs since 2013 (Table 1). All the procedures in this study were approved by the “Institutional Animal Care and Use Committee of National Taiwan University”, and no fish were sacrificed or injured.

**Table 1:** Samples collected in this study.

Port landings, fresh meat, (N = 231)			Fin products, dried or water-soaked (N = 316)				Custom-detained samples (N = 113)
Yilan	Taitung	Pingtung	Taipei	Taichung	Changhua	Kaohsiung	Kaohsiung
2013/6/4 NFA (22)	2013/3/18, XG (42)	2013/3/16, TK (26)	2012/10/7, HG (33)	2013/3/30, MY (23)	2013/9/9, LMY (29)	2013/9/21, SY (13)	2013/12/4 (5)
	2013/6/5, XG (38)	2013/8/6, TK (13)	2013/8/26, WC (28)	2013/6/6, NGF (26)		2014/6/9 DY, (11)	2013/12/9 (37)
	2014/6/26, XG (22)	2014/4/19, TK (63)	2014/6/13, SK (5)	*2013/7/25, NGF (31)			2014/3/12 (18)
	2014/6/27, XG (5)			2013/9/9, NGF (29)			2014/3/24 (23)
				2014/4/6, YL (46)			2014/5/3 (5)
				2014/5/5, JG (42)			2014/5/8 (8)
							2014/5/15 (3)
							2014/5/22 (8)
							2014/7/11 (6)

Number in the parenthesis is sample size. The sampling location or store was expressed as an abbreviation (NFA: NanFangAo Port; XG: XinGang Port; TK: TungKang Port; HG: HuGuang Medicine & Drug Ltd. Co. WC: Watsons the Chemist Ltd. Co.; SK: Shiitake King International Enterprise; MY: Ming Yuan Food Ltd. Co.; NGF: Natural Gold Food Ltd. Co.; YL: YongLi trading Co.; JG: JianGuo market; LMY: LiMingYu cooking oil Co.; SY: ShengYi Co.; DY: DaYi Co.).

* Water-soaked fin products.

### Shark species identification

We used the Chelex-resin method described by Holmes et al. (2009) [22] and the Geno*Plus* Genomic DNA Extraction Miniprep System (VIOGENE) to extract DNA from fresh tissues and fin products, respectively. To identify the species, we amplified the *cox1* gene with the use of primer pairs F1/R1 and F2/R2, as described by Ward et al. (2005) [23]. The reaction was carried out using TaKaRa *Ex Taq* (TaKaRa) and was run for 35 cycles at an annealing temperature of 54℃. The PCR products were sequenced in the Center for Biotechnology, National Taiwan University and the species of the samples were identified by comparing the sequences on the Barcode of Life Data Systems (BOLD systems, www.boldsystems.org) [24]. The species identification was based on the percentage of sequence identity, and we used a value higher than 99% as the general criterion for same-species confirmation. However if two or more species were simultaneously shown with high percentages of identity, the neighbor-joining tree was constructed in the BOLD systems and was used as an ancillary tool for identification. All the sequences identified in this study are available on GenBank in the accession numbers: KP719228—KP719887.

### Sequence analysis and geographical differentiation

The nucleotide composition and genetic distances of the sequences obtained in this study were calculated using Molecular Evolutionary Genetics Analysis (MEGA) 5.2.1 [25]. For the genetic distance analyses, we chose the TN93 substitution model for the intra- and inter-species distance calculation [26]. Because oceanic whitetip sharks (*C*. *longimanus*), thresher sharks (*Alopias pelagicus*, *A*. *superciliosus*, and *A*. *vulpinus*), and hammerhead sharks (*Sphyrna zygaena*, *S*. *mokarran*, and *S*. *lewini*) have been individually forbidden from catching in certain oceans, we also analyzed the *cox1* sequences of these sharks. Sequences derived from this study as well as sequences downloaded from the BOLD database were analyzed to examine whether certain molecular characteristics would be suitable for discriminating their origins. We also constructed phylogenetic trees by MEGA, using the maximum likelihood (ML) method with 1,000 bootstrap replications.

## Results

### Species identification and sequence analysis

**Fig 1:**
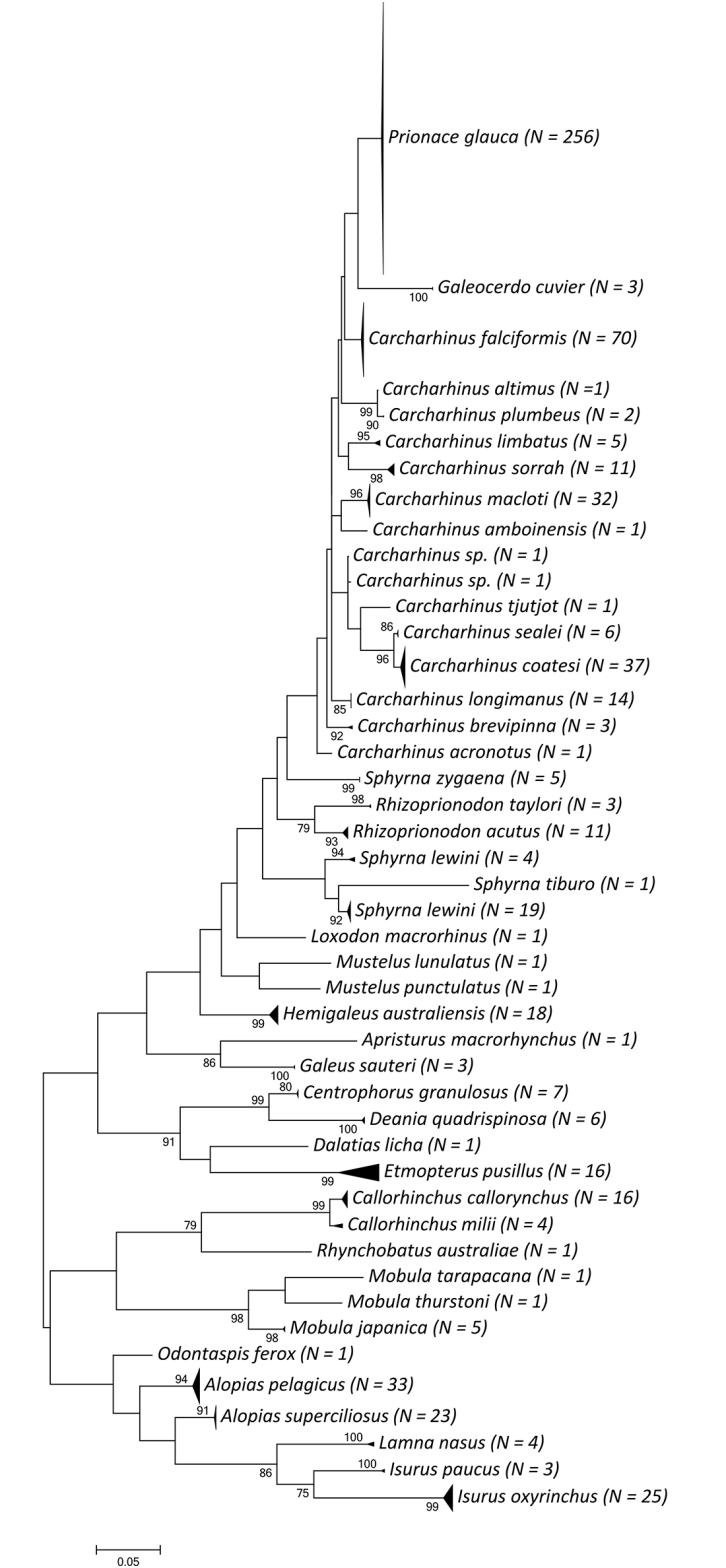
Maximum likelihood tree for all 660 sequences obtained in this study. Each species formed a distinct group in the tree, indicating the robustness of the DNA barcoding method on shark species identification. The tree was constructed with 1,000 bootstrap replications and only bootstrap values higher than 70% are shown on the branch.

In total we identified 23 species (in 10 families) from the 231 port landings sample, 24 species (in six families) from the 316 shark fin products (Table 2), and 14 species (in five families) from the 113 detained shark fins provided by Kaohsiung customs (Table 3). The average length of these 660 sequences was 523 ± 69 bp and the average nucleotide composition was T: 34.3%, C: 24.8%, A: 26.6%, and G: 14.3%. The TN93 distance within species was 0.005 ± 0.003, while distances between species were 0.074 ± 0.03 and 0.154 ± 0.054 within genus and family, respectively. Most of the samples could be robustly identified by either the BOLD identification system or the neighbor-joining tree and formed distinct groups when analyzed together (Fig 1). Since no species-specific characteristic in the *cox1* gene could distinguish *Carcharhinus obscurus* from *C*. *galapagensis*, two individuals were expressed as *Carcharhinus* sp.

**Table 2:** Species identified in port landings sample and fin products sample.

Port landings/species	N (%)	IUCN	Fin products/species	N (%)	IUCN
^a^***Prionace glauca***	109 (47.2)	NT	^a^***Prionace glauca***	106 (33.5)	NT
^a^***Carcharhinus falciformis***	18 (7.8)	NT	^a^***Carcharhinus falciformis***	43 (13.6)	NT
^a^***Alopias superciliosus***	17 (7.4)	VU	^c^*Carcharhinus coatesi*	37 (11.7)	NE
^a^***Alopias pelagicus***	16 (6.9)	VU	*Carcharhinus macloti*	32 (10.1)	NT
*Etmopterus pusillus*	16 (6.9)	LC	^a^***Sphyrna lewini***	20 (6.3)	EN
^a^***Isurus oxyrinchus***	13 (5.6)	VU	^c^*Hemigaleus australiensis*	18 (5.7)	LC
*Centrophorus granulosus*	7 (3.0)	VU	***Carcharhinus sorrah***	9 (2.8)	NT
^a^*Deania quadrispinosa*	6 (2.6)	NT	*Carcharhinus sealei*	6 (1.9)	NT
*Mobula japonica*	5 (2.2)	NT	*Rhizoprionodon acutus*	6 (1.9)	LC
^a^*Sphyrna zygaena*	5 (2.2)	VU	^a^***Alopias pelagicus***	5 (1.6)	VU
*Galeus sauteri*	3 (1.3)	DD	^a^*Carcharhinus limbatus*	5 (1.6)	NT
^a^***Sphyrna lewini***	3 (1.3)	EN	^a^***Isurus oxyrinchus***	5 (1.6)	VU
^b^*Carcharhinus* sp.	2 (0.9)	VU	^a^***Carcharhinus longimanus***	4 (1.3)	VU
^a^*Carcharhinus plumbeus*	2 (0.9)	VU	^c^*Lamna nasus*	4 (1.3)	VU
*Apristurus macrorhynchus*	1 (0.4)	DD	^a^*Carcharhinus brevipinna*	3 (0.9)	NT
^a^***Carcharhinus longimanus***	1 (0.4)	NT	*Rhizoprionodon taylori*	3 (0.9)	LC
***Carcharhinus sorrah***	1 (0.4)	NT	^a^***Alopias superciliosus***	2 (0.6)	VU
*Dalatias licha*	1 (0.4)	NT	^a^***Galeocerdo cuvier***	2 (0.6)	VU
^a^***Galeocerdo cuvier***	1 (0.4)	VU	*Carcharhinus altimus*	1 (0.3)	DD
*Isurus paucus*	1 (0.4)	VU	*Carcharhinus amboinensis*	1 (0.3)	DD
*Mobula tarapacana*	1 (0.4)	DD	*Carcharhinus tjutjot*	1 (0.3)	DD
*Mobula thurstoni*	1 (0.4)	NT	*Loxodon macrorhinus*	1 (0.3)	LC
*Odontaspis ferox*	1 (0.4)	VU	*Rhynchobatus australiae*	1 (0.3)	VU
			^c^*Sphyrna tiburo*	1 (0.3)	LC
Total	231		Total	316	

The identified species were ranked according to the sample number, with the percentages shown in the parentheses. The conservation status of the identified species was expressed as an abbreviation (EN: endangered; VU: vulnerable; NT: near threatened; LC: least concern; DD: data deficient) and a bold letter highlighted species present in both the port landings and fin products.

^a^ Species also presented in the shark meats examined in Liu et al. (2013).

^b^ Only the genus was identified; The conservation status was assigned based on *Carcharhinus obscurus*.

^c^ Species not found in Taiwan’s waters.

**Table 3:** Species identified in customs-detained samples.

Customs-detained/species	N (%)	IUCN
***Prionace glauca***	41 (36.3)	NT
^a^*Callorhinchus callorynchus*	16 (14.2)	LC
***Alopias pelagicus***	12 (10.6)	VU
***Carcharhinus falciformis***	9 (8.0)	NT
***Carcharhinus longimanus***	9 (8.0)	VU
***Isurus oxyrinchus***	7 (6.2)	VU
*Rhizoprionodon acutus*	5 (4.4)	LC
***Alopias superciliosus***	4 (3.5)	VU
^a^*Callorhinchus milii*	4 (3.5)	LC
***Isurus paucus***	2 (1.8)	VU
^a^*Carcharhinus acronotus*	1 (0.9)	NT
***Carcharhinus sorrah***	1 (0.9)	NT
^a^*Mustelus lunulatus*	1 (0.9)	LC
^a^*Mustelus punctulatus*	1 (0.9)	DD
Total	113	

The identified species were ranked according to the sample number, with the percentages shown in the parentheses. The conservation status of the identified species was expressed as an abbreviation (EN: endangered; VU: vulnerable; NT: near threatened; LC: least concern; DD: data deficient) and a bold letter highlighted species present in either the port landings or fin products.

^a^ Species not found in Taiwan’s waters.

### Species composition and conservation status

For the species composition, the blue shark (*P*. *glauca*) was the most common species, comprising 47.2% of the port landings sample, 33.5% of the fin products, and 36.3% of the customs sample. The silky shark *C*. *falciformis* and the elephantfish *Callorhinchus callorynchus* were ranked behind the blue shark. The port landings and fin products had nine common species, compromising 77.5% (N = 179) and 62.0% (N = 196), respectively. For the customs sample, eight of the 14 identified species (N = 85, 75.2%) could also be seen in the port landings sample. All the species identified in the port landings are known species in Taiwan’s waters. While in the fin products and customs samples, four species (*Carcharhinus coatesi*, *Hemigaleus australiensis*, *Lamna nasus*, and *Sphyrna tiburo*) and five species (*Callorhinchus callorynchus*, *C*. *milii*, *Carcharhinus acronotus*, *Mustelus lunulatus*, and *M*. *punctulatus*) are not found in Taiwan’s waters or even in the Western Pacific Ocean, respectively.

Among all the species identified (N = 43), an endangered species (*S*. *lewini*) according to the IUCN Red List (www.iucnredlist.org) was found. The remains were 13 vulnerable species, 12 near threatened species, nine least concerned species, and eight species that were data deficient or not evaluated (Tables 2 and 3). Specimens categorized as threatened (endangered and vulnerable species) accounted for 22.1% (N = 146) of all the samples.

### Geographical differentiation

**Fig 2:**
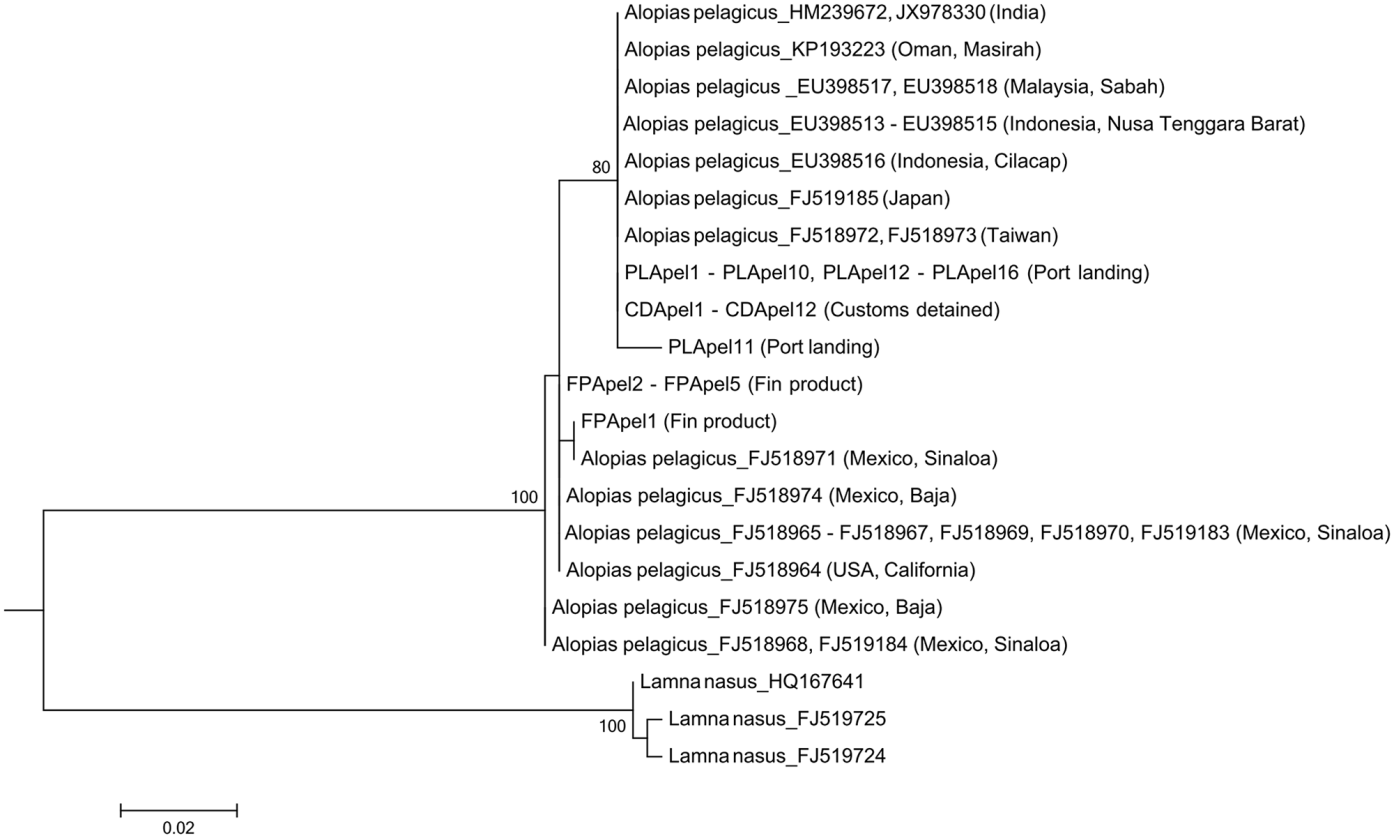
Maximum likelihood tree for the pelagic thresher shark, *Alopias pelagicus*. The tree was constructed with a 403 bp *cox1* gene fragment and ran for 1,000 bootstrap replications. DNA sequences of the sharks from the Indo-West Pacific Ocean can be distinguished from those of the East Pacific Ocean. The sequences from the database were expressed as accession numbers and our specimens as label abbreviations (PL, FP, and CD for port landings, fin products, and customs-detained samples, respectively). Different sequences with the same haplotype were placed in a single taxon (with dashes to connect consecutive sequences and commas to separate disjunctive ones). Only bootstrap values higher than 70% are shown on the branch.

Among the seven species examined for geographical differentiation, only the pelagic thresher shark (*A*. *pelagicus*), the common thresher shark (*A*. *vulpinus*), the smooth hammerhead shark (*S*. *zygaena*), and the scalloped hammerhead shark (*S*. *lewini*) showed differentiation among different oceans. The ML tree of the pelagic thresher shark showed that Indo-West Pacific individuals could be distinguished from East Pacific individuals, with all the *A*. *pelagicus* in our port landings and customs-detained samples placed in the former group and the five in fin products sample in the latter one (Fig 2).

**Fig 3:**
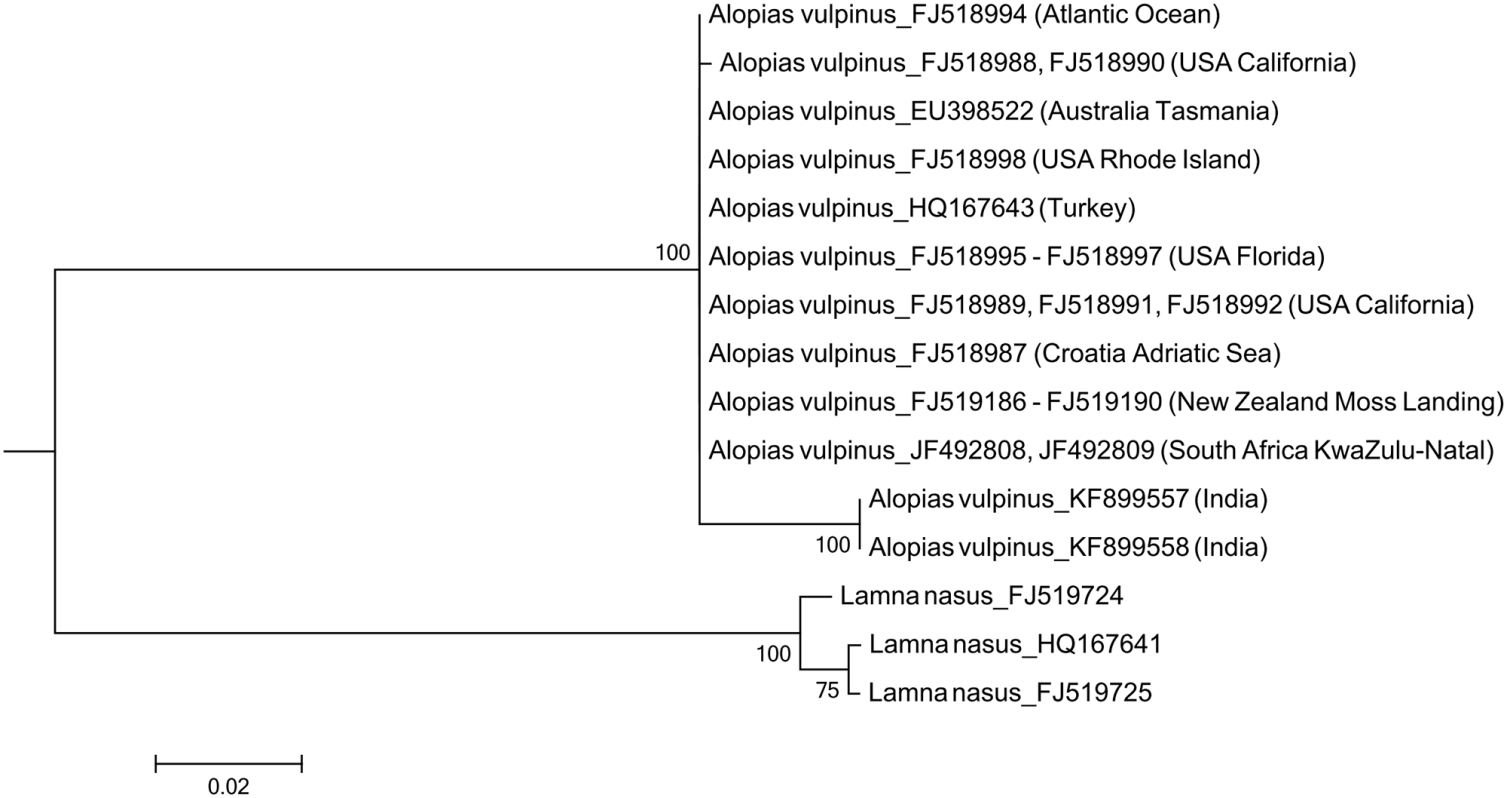
Maximum likelihood tree for the common thresher shark, *Alopias vulpinus*. The tree was constructed with a 651 bp *cox1* gene fragment and ran for 1,000 bootstrap replicateion. DNA sequences of the sharks from the Indian Ocean and those from other regions form two distinct groups. Only bootstrap values higher than 70% are shown on the branch.

For the common thresher shark, individuals from the Indian Ocean could be separated from individuals in other ocean basins (Fig 3 - *A*. *vulpinus* was not identified in this study). Remarkably, four *A*. *vulpinus* sequences in the BOLD systems reported to have been collected from Indian Ocean (accession number: FJ347901 –FJ347904) were identical to those of *A*. *superciliosus*. We thereby omitted the four sequences from the analysis as they might have been *A*. *superciliosus* individuals misidentified as *A*. *vulpinus*.

**Fig 4:**
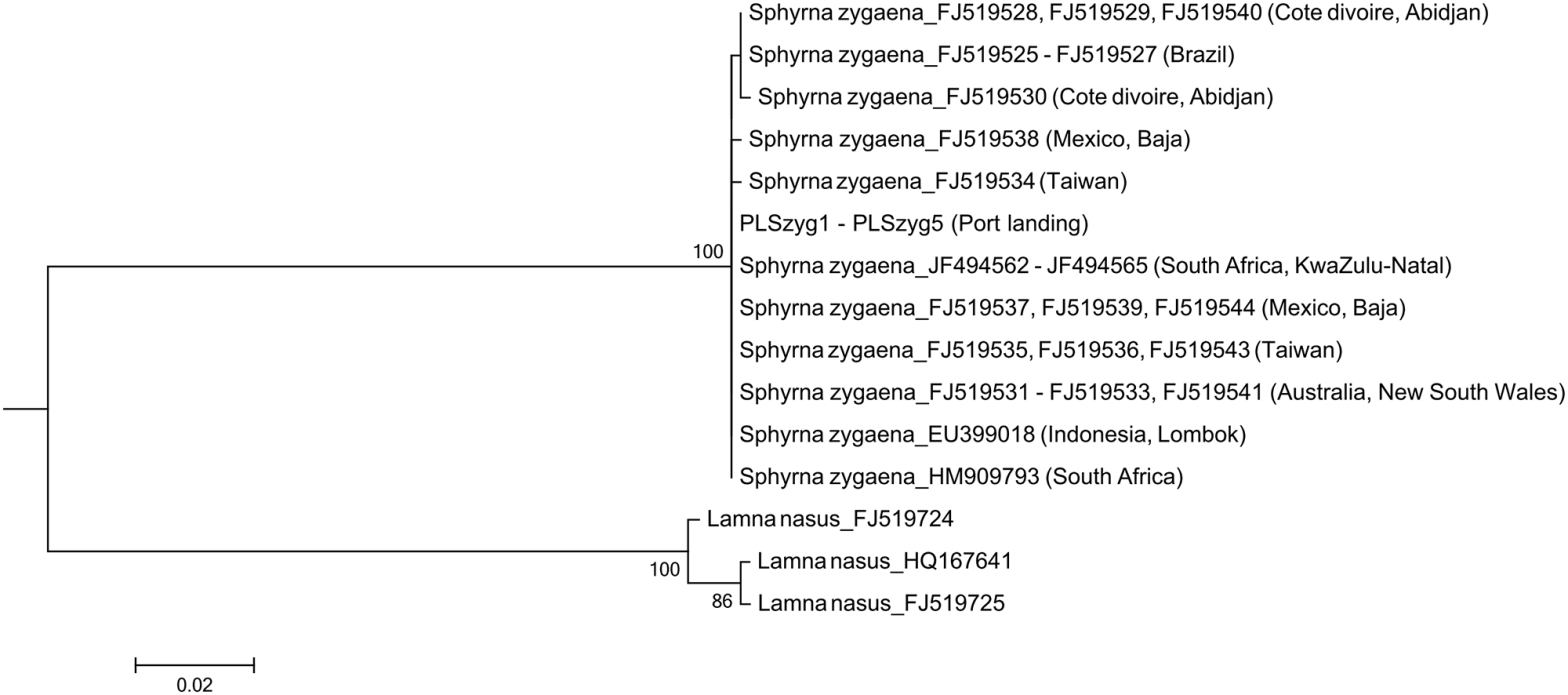
Maximum likelihood tree for the smooth hammerhead shark, *Sphyrna zygaena*. The tree was constructed with a 403 bp *cox1* gene fragment and ran for 1,000 bootstrap replications. DNA sequences of the sharks from the Atlantic Ocean can be distinguished from those of other regions. Only bootstrap values higher than 70% are shown on the branch.

For the smooth hammerhead shark, one single nucleotide polymorphism (SNP) was found in the sequences. The individuals that had been collected from the Atlantic Ocean formed a distinct group that separated from other Indo-Pacific individuals, with the five *S*. *zygaena* individuals in our port landings sample being placed in the latter group (Fig 4).

**Fig 5:**
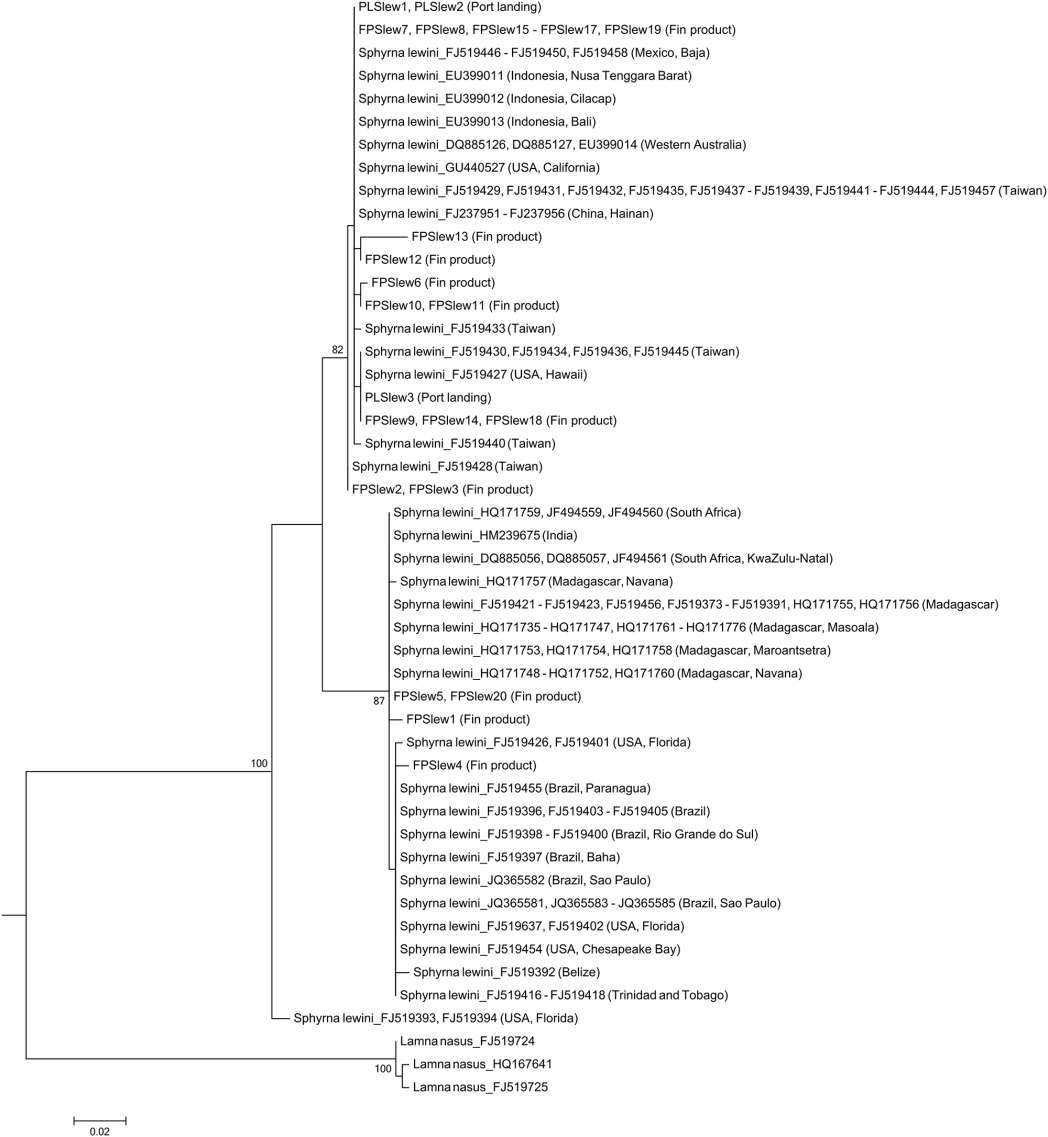
Maximum likelihood tree for the scalloped hammerhead shark, *Sphyrna lewini*. The tree was constructed with a 652 bp *cox1* gene fragment and ran for 1,000 bootstrap replications. DNA sequences of the sharks can distinguish those from the Pacific, Atlantic, and Indian Oceans except for two sequences from Florida, (FJ519393 and FJ519394), which form a distinct group sister to all other sequences of *S*. *lewini*. Only bootstrap values higher than 70% are shown on the branch.

Regarding the scalloped hammerhead shark, individuals from the three major ocean basins (i.e., the Pacific, Atlantic, and Indian Oceans) could be specifically identified as three groups in the phylogenetic tree (except for two Atlantic individuals, which were placed at the base position of the ML tree). The phylogenetic result also indicated that the three *S*. *lewini* in our port landings sample were caught in the Pacific Ocean. Among the 20 *S*. *lewini* fin products, 16 of them came from the Pacific Ocean, three from the Indian Ocean, and one from the Atlantic Ocean (Fig 5).

For the remaining three species (i.e., *C*. *longimanus*, *A*. *superciliosus*, and *S*. *mokarran*), the phylogenetic results demonstrated either no variation in the *cox1* gene or no clear differentiation pattern in corresponding to geographical boundary.

## Discussion

### Species composition and conservation implication

Overall, 62% of the species in the fin products also appeared in the port landings sample, consistent with the observation that the majority of fin products in Taiwan comes from Taiwanese domestic fisheries. Although most of these sharks have extensive ranges, the fin products in Taiwan’s market seem dominated more by domestic supplies than by international sources, as evident from the 20 scalloped hammerhead sharks in fin products, of which 80% came from the Pacific Ocean.

However, the international trade of shark fins also contributes significantly to Taiwan’s market. Thirteen of the 24 species found in the fin products are neither present in our port landings sample nor in the report of Liu et al. (2013) [19], and four of them are even unobserved in Taiwan’s waters. These species are most likely a result of international trade. In addition, some domestic-accessible species might also have international sources. For instance, although the pelagic thresher shark can be found in Taiwan’s waters, all the five *A*. *pelagicus* identified in fin products were exclusively East-Pacific in origin and must be imported. The customs sample further supports this with over three-fourths of the sample came from species that are also caught by domestic fisheries. Because these samples were detained for not being declared while being imported, it is implied that certain portion of the domestic-available shark fins comes from international source instead. The contribution of international trade in Taiwan’s shark fin markets is therefore possibly underestimated.

The dominant species (*P*. *glauca*) in this study is categorized as a near-threatened species in the IUCN Red List, and population declines in this species have been reported since the 1980s [9, 27, 28]. These population declines might have resulted from the high by-catch rate and the rapid expansion of directed fisheries. Because of its cosmopolitan distribution, the blue shark might confound the efforts of the marine reserves, just as other highly migratory species do. Placement of regulations on fishing efforts might, as an alternative, be useful to reduce fishing pressure [9, 27].

Among the 43 species identified in this study, 14 are categorized as threatened species in the IUCN Red List. This high proportion might be attributed to the shark-bycatching nature of long-line fisheries and to the lack of conservation concern among fishers. A modification of fishing gears might prove beneficial in increasing the survival rates of the by-catch sharks [29]. Considering the ecological roles and the large annual landings of these sharks, the improved management of these resources is necessary, and global or ocean-based regulations, like those for thresher sharks and hammerhead sharks, are likely for other sharks in the future. Regarding this, the application of DNA barcoding techniques to shark identification [22, 23, 30] facilitates a rapid and accurate species identification, which is essential for enforcing regulations.

To thoroughly implement trading regulations and prevent smuggling, Taiwan’s customs agency has collaborated with our lab to identify the detained shark fins since 2013. The result in this study revealed five threatened species, with two of them falling under regional catching regulations (*A*. *pelagicus* and *A*. *superciliosus*). Unfortunately, our sequence data can only identify the Indo-West Pacific origin of the *A*. *pelagicus* specimens and fail to judge the legality of these fish catches on the regulation criteria of RFMOs.

### Geographical differentiation of sharks

In this study we showed that the pelagic thresher shark, the common thresher shark, the smooth hammerhead shark, and the scalloped hammerhead shark displayed clear regional differentiations in their *cox1* sequences. According to the regulation of the International Commission for the Conservation of Atlantic Tunas (ICCAT) and the Indian Ocean Tuna Commission (IOTC), the catching of hammerhead sharks (except for *Sphyrna tiburo*) is prohibited in the Atlantic Ocean, as is the catch of thresher sharks in both the Atlantic and Indian oceans. Unfortunately, the differentiation patterns of the two thresher sharks do not correspond to the fishery-restricted regions, thereby making the judgment of an illegal catch difficult. The differentiation of the common thresher shark is even less convincing because only two representatives of the Indian population were available for this study.

Previous studies have extensively expounded on the population structure of scalloped hammerhead sharks [31–33]. Similar to the result of Naylor et al. (2012), our result shows that the Atlantic population of *S*. *lewini* can be distinguished from other populations by the *cox1* gene [30]. The fact that the two reference sequences from Florida (FJ519393 and FJ519394) were not grouped in the main Atlantic cluster in our phylogenetic tree does not diminish the applicability of this gene marker on identifying the origins of *S*. *lewini* individuals. Because the two sequences were distinct from all other *S*. *lewini* individuals, they likely represent the cryptic *Sphyrna* species in the West Atlantic Ocean, as assumed in previous studies [32, 34]. For the smooth hammerhead shark, although the *nadh2* gene used by Naylor et al. (2012) yielded no geographical differentiation [30], the Atlantic *S*. *zygaena* can be distinguished by the *cox1* gene in this study. Remarkably, even though we did not find geographical differentiation in the *cox1* gene of the great hammerhead shark, the Atlantic *S*. *mokarran* was found able to be clearly distinguished by the *nadh2* gene [30]. These complementary results indicate that both the species and the oceanic origin of these sharks can be simultaneously identified by the DNA barcoding approaches. Because our results imply that *S*. *lewini* is still being fished in the Atlantic Ocean, we conclude that the creation and enforcement of more stringent regulation on the trade and catch of this species is an urgent issue.

### An identification ambiguity in BOLD systems

Although many reports have declared the applicability of DNA barcoding on shark species identification [22, 23, 30], some ambiguities in the BOLD systems still need to be carefully treated. As demonstrated in a previous report [8], two individuals in this study were identified as either *Carcharhinus obscurus* or *C*. *galapagensis*. Although Garrick (1982) and Nalyor (1992) [35, 36] had claimed the taxonomical robustness of *C*. *obscurus* and *C*. *galapagensis*, these two species could not be distinguished by either DNA barcoding or the nucleotide diagnostic approach [8, 30]. Our analysis showed that the two species shared the same SNPs in the *cox1* sequence. One explanation is that these SNPs are plesiomorphies inherited from a recent common ancestor. However, as suggested by Wong et al. (2009), species hybridization is another possibility: the distributions of these two species overlap and no solid evidence of reproductive isolation exists [8]. Based on the molecular evidence from the *nadh2* gene, Naylor et al. (2012) even challenged the taxon integrity of *C*. *galapagensis* and suggested that *C*. *galapagensis* might merely be the oceanic form of *C*. *obscurus* [30]. Because we cannot distinguish these two species by either *cox1* or the *nadh2* gene, we should develop a more discriminating DNA marker, at least until strong evidence arises to support the synonymy of these two species.

## Supporting Information

S1 Table List of all the 660 samples collected in this study. (DOCX)
