# Supplementary material for: The Species and Origin of Shark Fins in Taiwan’s Fishing Ports, Markets, and Customs Detention: A DNA Barcoding Analysis
Source: PLoS One. 2016 Jan 22;11(1):e0147290. doi: 10.1371/journal.pone.0147290 (PMC4723227; doi:10.1371/journal.pone.0147290)
Supplement: S1 Table — (DOCX) [file pone.0147290.s001.docx]

Supporting Information

Legend to supplementary table.

**Table S1. List of all the 660 samples collected in this study.**

The samples were named according to the sources of the samples (PL: port landings; FP: fin products; CD: customs-detained) and the species that being identified. GenBank accession number for each sample was provided. Sampling locations were giving as abbreviation (NFA: NanFangAo Port; XG: XinGang Port; TK: TungKang Port; HG: HuGuang Medicine & Drug Ltd. Co. WC: Watsons the Chemist Ltd. Co.; SK: Shiitake King International Enterprise; MY: Ming Yuan Food Ltd. Co.; NGF: Natural Gold Food Ltd. Co.; YL: YongLi trading Co.; JG: JianGuo market; LMY: LiMingYu cooking oil Co.; SY: ShengYi Co.; DY: DaYi Co.). For the fin products sample, Weight (g) and shape were also provided except for the water-soaked fin products sample (shape 1: right triangle; shape 2: equilateral triangle; shape 3: irregular shapes).

**Table S1. List of all the 660 samples collected in this study.**

| Code | Accession # | Species | Location | Date | Weight (g) | Shape |
| --- | --- | --- | --- | --- | --- | --- |
| PLDqua1 | KP719228 | *Deania quadrispinosa* | XG | 2014/6/26 | - | - |
| PLDqua2 | KP719229 | *Deania quadrispinosa* | XG | 2014/6/26 | - | - |
| PLDqua3 | KP719230 | *Deania quadrispinosa* | XG | 2014/6/26 | - | - |
| PLDqua4 | KP719231 | *Deania quadrispinosa* | XG | 2014/6/26 | - | - |
| PLDqua5 | KP719232 | *Deania quadrispinosa* | XG | 2014/6/26 | - | - |
| PLDqua6 | KP719233 | *Deania quadrispinosa* | XG | 2014/6/27 | - | - |
| PLIoxy1 | KP719234 | *Isurus oxyrinchus* | TK | 2014/4/19 | - | - |
| PLIoxy2 | KP719235 | *Isurus oxyrinchus* | TK | 2014/4/19 | - | - |
| PLIoxy3 | KP719236 | *Isurus oxyrinchus* | XG | 2013/6/5 | - | - |
| PLIoxy4 | KP719237 | *Isurus oxyrinchus* | XG | 2013/6/5 | - | - |
| PLIoxy5 | KP719238 | *Isurus oxyrinchus* | XG | 2013/6/5 | - | - |
| PLIoxy6 | KP719239 | *Isurus oxyrinchus* | NFA | 2013/6/4 | - | - |
| PLIoxy7 | KP719240 | *Isurus oxyrinchus* | XG | 2013/3/18 | - | - |
| PLIoxy8 | KP719241 | *Isurus oxyrinchus* | XG | 2013/3/18 | - | - |
| PLIoxy9 | KP719242 | *Isurus oxyrinchus* | XG | 2013/3/18 | - | - |
| PLIoxy10 | KP719243 | *Isurus oxyrinchus* | XG | 2013/3/18 | - | - |
| PLIoxy11 | KP719244 | *Isurus oxyrinchus* | XG | 2013/3/18 | - | - |
| PLIoxy12 | KP719245 | *Isurus oxyrinchus* | XG | 2013/3/18 | - | - |
| PLIoxy13 | KP719246 | *Isurus oxyrinchus* | XG | 2013/3/18 | - | - |
| PLIpau1 | KP719247 | *Isurus paucus* | TK | 2013/3/16 | - | - |
| PLApel1 | KP719248 | *Alopias pelagicus* | XG | 2013/6/5 | - | - |
| PLApel2 | KP719249 | *Alopias pelagicus* | XG | 2013/6/5 | - | - |
| PLApel3 | KP719250 | *Alopias pelagicus* | XG | 2013/6/5 | - | - |
| PLApel4 | KP719251 | *Alopias pelagicus* | XG | 2013/6/5 | - | - |
| PLApel5 | KP719252 | *Alopias pelagicus* | XG | 2013/6/5 | - | - |
| PLApel6 | KP719253 | *Alopias pelagicus* | XG | 2013/6/5 | - | - |
| PLApel7 | KP719254 | *Alopias pelagicus* | XG | 2013/6/5 | - | - |
| PLApel8 | KP719255 | *Alopias pelagicus* | XG | 2013/6/5 | - | - |
| PLApel9 | KP719256 | *Alopias pelagicus* | XG | 2013/3/18 | - | - |
| PLApel10 | KP719257 | *Alopias pelagicus* | XG | 2013/3/18 | - | - |
| PLApel11 | KP719258 | *Alopias pelagicus* | XG | 2013/3/18 | - | - |
| PLApel12 | KP719259 | *Alopias pelagicus* | XG | 2013/3/18 | - | - |
| PLApel13 | KP719260 | *Alopias pelagicus* | XG | 2013/3/18 | - | - |
| PLApel14 | KP719261 | *Alopias pelagicus* | TK | 2013/3/16 | - | - |
| PLApel15 | KP719262 | *Alopias pelagicus* | TK | 2013/3/16 | - | - |
| PLApel16 | KP719263 | *Alopias pelagicus* | TK | 2013/3/16 | - | - |
| PLAsup1 | KP719264 | *Alopias superciliosus* | XG | 2013/6/5 | - | - |
| PLAsup2 | KP719265 | *Alopias superciliosus* | XG | 2013/6/5 | - | - |
| PLAsup3 | KP719266 | *Alopias superciliosus* | NFA | 2013/6/4 | - | - |
| PLAsup4 | KP719267 | *Alopias superciliosus* | NFA | 2013/6/4 | - | - |

Continued

| Code | Accession # | Species | Location | Date | Weight (g) | Shape |
| --- | --- | --- | --- | --- | --- | --- |
| PLAsup5 | KP719268 | *Alopias superciliosus* | NFA | 2013/6/4 | - | - |
| PLAsup6 | KP719269 | *Alopias superciliosus* | NFA | 2013/6/4 | - | - |
| PLAsup7 | KP719270 | *Alopias superciliosus* | NFA | 2013/6/4 | - | - |
| PLAsup8 | KP719271 | *Alopias superciliosus* | XG | 2013/3/18 | - | - |
| PLAsup9 | KP719272 | *Alopias superciliosus* | XG | 2013/3/18 | - | - |
| PLAsup10 | KP719273 | *Alopias superciliosus* | XG | 2013/3/18 | - | - |
| PLAsup11 | KP719274 | *Alopias superciliosus* | XG | 2013/3/18 | - | - |
| PLAsup12 | KP719275 | *Alopias superciliosus* | XG | 2013/3/18 | - | - |
| PLAsup13 | KP719276 | *Alopias superciliosus* | TK | 2013/3/16 | - | - |
| PLAsup14 | KP719277 | *Alopias superciliosus* | TK | 2013/3/16 | - | - |
| PLAsup15 | KP719278 | *Alopias superciliosus* | TK | 2013/3/16 | - | - |
| PLAsup16 | KP719279 | *Alopias superciliosus* | TK | 2013/3/16 | - | - |
| PLAsup17 | KP719280 | *Alopias superciliosus* | TK | 2013/3/16 | - | - |
| PLAmac1 | KP719281 | *Apristurus macrorhynchus* | XG | 2014/6/27 | - | - |
| PLCfal1 | KP719282 | *Carcharhinus falciformis* | XG | 2013/6/5 | - | - |
| PLCfal2 | KP719283 | *Carcharhinus falciformis* | XG | 2013/6/5 | - | - |
| PLCfal3 | KP719284 | *Carcharhinus falciformis* | TK | 2014/4/19 | - | - |
| PLCfal4 | KP719285 | *Carcharhinus falciformis* | XG | 2013/3/18 | - | - |
| PLCfal5 | KP719286 | *Carcharhinus falciformis* | XG | 2013/3/18 | - | - |
| PLCfal6 | KP719287 | *Carcharhinus falciformis* | TK | 2013/3/16 | - | - |
| PLCfal7 | KP719288 | *Carcharhinus falciformis* | TK | 2013/3/16 | - | - |
| PLCfal8 | KP719289 | *Carcharhinus falciformis* | TK | 2013/3/16 | - | - |
| PLCfal9 | KP719290 | *Carcharhinus falciformis* | TK | 2013/3/16 | - | - |
| PLCfal10 | KP719291 | *Carcharhinus falciformis* | TK | 2013/3/16 | - | - |
| PLCfal11 | KP719292 | *Carcharhinus falciformis* | TK | 2013/3/16 | - | - |
| PLCfal12 | KP719293 | *Carcharhinus falciformis* | TK | 2013/3/16 | - | - |
| PLCfal13 | KP719294 | *Carcharhinus falciformis* | TK | 2013/3/16 | - | - |
| PLCfal14 | KP719295 | *Carcharhinus falciformis* | TK | 2013/3/16 | - | - |
| PLCfal15 | KP719296 | *Carcharhinus falciformis* | TK | 2013/3/16 | - | - |
| PLCfal16 | KP719297 | *Carcharhinus falciformis* | TK | 2013/3/16 | - | - |
| PLCfal17 | KP719298 | *Carcharhinus falciformis* | TK | 2013/8/6 | - | - |
| PLCfal18 | KP719299 | *Carcharhinus falciformis* | TK | 2013/8/6 | - | - |
| PLClon1 | KP719300 | *Carcharhinus longimanus* | TK | 2013/8/6 | - | - |
| PLCobs1 | KP719301 | *Carcharhinus obscurus* | XG | 2013/3/18 | - | - |
| PLCobs2 | KP719302 | *Carcharhinus obscurus* | TK | 2013/3/16 | - | - |
| PLCplu1 | KP719303 | *Carcharhinus plumbeus* | XG | 2013/6/5 | - | - |
| PLCplu2 | KP719304 | *Carcharhinus plumbeus* | NFA | 2013/6/4 | - | - |
| PLCsor1 | KP719305 | *Carcharhinus sorrah* | XG | 2013/6/5 | - | - |
| PLCgra1 | KP719306 | *Centrophorus granulosus* | XG | 2013/3/18 | - | - |
| PLCgra2 | KP719307 | *Centrophorus granulosus* | XG | 2013/3/18 | - | - |

Continued

| Code | Accession # | Species | Location | Date | Weight (g) | Shape |
| --- | --- | --- | --- | --- | --- | --- |
| PLCgra3 | KP719308 | *Centrophorus granulosus* | XG | 2013/3/18 | - | - |
| PLCgra4 | KP719309 | *Centrophorus granulosus* | XG | 2013/3/18 | - | - |
| PLCgra5 | KP719310 | *Centrophorus granulosus* | XG | 2013/3/18 | - | - |
| PLCgra6 | KP719311 | *Centrophorus granulosus* | XG | 2013/3/18 | - | - |
| PLCgra7 | KP719312 | *Centrophorus granulosus* | XG | 2013/3/18 | - | - |
| PLDlic1 | KP719313 | *Dalatias licha* | XG | 2014/6/26 | - | - |
| PLEpus1 | KP719314 | *Etmopterus pusillus* | XG | 2014/6/26 | - | - |
| PLEpus2 | KP719315 | *Etmopterus pusillus* | XG | 2014/6/26 | - | - |
| PLEpus3 | KP719316 | *Etmopterus pusillus* | XG | 2014/6/26 | - | - |
| PLEpus4 | KP719317 | *Etmopterus pusillus* | XG | 2014/6/26 | - | - |
| PLEpus5 | KP719318 | *Etmopterus pusillus* | XG | 2014/6/26 | - | - |
| PLEpus6 | KP719319 | *Etmopterus pusillus* | XG | 2014/6/26 | - | - |
| PLEpus7 | KP719320 | *Etmopterus pusillus* | XG | 2014/6/26 | - | - |
| PLEpus8 | KP719321 | *Etmopterus pusillus* | XG | 2014/6/26 | - | - |
| PLEpus9 | KP719322 | *Etmopterus pusillus* | XG | 2014/6/26 | - | - |
| PLEpus10 | KP719323 | *Etmopterus pusillus* | XG | 2014/6/26 | - | - |
| PLEpus11 | KP719324 | *Etmopterus pusillus* | XG | 2014/6/26 | - | - |
| PLEpus12 | KP719325 | *Etmopterus pusillus* | XG | 2014/6/26 | - | - |
| PLEpus13 | KP719326 | *Etmopterus pusillus* | XG | 2014/6/26 | - | - |
| PLEpus14 | KP719327 | *Etmopterus pusillus* | XG | 2014/6/26 | - | - |
| PLEpus15 | KP719328 | *Etmopterus pusillus* | XG | 2014/6/26 | - | - |
| PLEpus16 | KP719329 | *Etmopterus pusillus* | XG | 2014/6/26 | - | - |
| PLGcuv1 | KP719330 | *Galeocerdo cuvier* | XG | 2013/3/18 | - | - |
| PLGsau1 | KP719331 | *Galeus sauteri* | XG | 2014/6/27 | - | - |
| PLGsau2 | KP719332 | *Galeus sauteri* | XG | 2014/6/27 | - | - |
| PLGsau3 | KP719333 | *Galeus sauteri* | XG | 2014/6/27 | - | - |
| PLMjap1 | KP719334 | *Mobula japanica* | XG | 2013/6/5 | - | - |
| PLMjap2 | KP719335 | *Mobula japanica* | XG | 2013/6/5 | - | - |
| PLMjap3 | KP719336 | *Mobula japanica* | XG | 2013/3/18 | - | - |
| PLMjap4 | KP719337 | *Mobula japanica* | XG | 2013/3/18 | - | - |
| PLMjap5 | KP719338 | *Mobula japanica* | XG | 2013/3/18 | - | - |
| PLMtar1 | KP719339 | *Mobula tarapacana* | XG | 2013/6/5 | - | - |
| PLMthu1 | KP719340 | *Mobula thurstoni* | XG | 2013/3/18 | - | - |
| PLOfer1 | KP719341 | *Odontaspis ferox* | XG | 2013/3/18 | - | - |
| PLPgla1 | KP719342 | *Prionace glauca* | XG | 2013/6/5 | - | - |
| PLPgla2 | KP719343 | *Prionace glauca* | XG | 2013/6/5 | - | - |
| PLPgla3 | KP719344 | *Prionace glauca* | XG | 2013/6/5 | - | - |
| PLPgla4 | KP719345 | *Prionace glauca* | XG | 2013/6/5 | - | - |
| PLPgla5 | KP719346 | *Prionace glauca* | XG | 2013/6/5 | - | - |
| PLPgla6 | KP719347 | *Prionace glauca* | XG | 2013/6/5 | - | - |

Continued

| Code | Accession # | Species | Location | Date | Weight (g) | Shape |
| --- | --- | --- | --- | --- | --- | --- |
| PLPgla7 | KP719348 | *Prionace glauca* | XG | 2013/6/5 | - | - |
| PLPgla8 | KP719349 | *Prionace glauca* | XG | 2013/6/5 | - | - |
| PLPgla9 | KP719350 | *Prionace glauca* | XG | 2013/6/5 | - | - |
| PLPgla10 | KP719351 | *Prionace glauca* | XG | 2013/6/5 | - | - |
| PLPgla11 | KP719352 | *Prionace glauca* | XG | 2013/6/5 | - | - |
| PLPgla12 | KP719353 | *Prionace glauca* | TK | 2014/4/19 | - | - |
| PLPgla13 | KP719354 | *Prionace glauca* | TK | 2014/4/19 | - | - |
| PLPgla14 | KP719355 | *Prionace glauca* | TK | 2014/4/19 | - | - |
| PLPgla15 | KP719356 | *Prionace glauca* | TK | 2014/4/19 | - | - |
| PLPgla16 | KP719357 | *Prionace glauca* | TK | 2014/4/19 | - | - |
| PLPgla17 | KP719358 | *Prionace glauca* | TK | 2014/4/19 | - | - |
| PLPgla18 | KP719359 | *Prionace glauca* | TK | 2014/4/19 | - | - |
| PLPgla19 | KP719360 | *Prionace glauca* | TK | 2014/4/19 | - | - |
| PLPgla20 | KP719361 | *Prionace glauca* | TK | 2014/4/19 | - | - |
| PLPgla21 | KP719362 | *Prionace glauca* | TK | 2014/4/19 | - | - |
| PLPgla22 | KP719363 | *Prionace glauca* | TK | 2014/4/19 | - | - |
| PLPgla23 | KP719364 | *Prionace glauca* | TK | 2014/4/19 | - | - |
| PLPgla24 | KP719365 | *Prionace glauca* | TK | 2014/4/19 | - | - |
| PLPgla25 | KP719366 | *Prionace glauca* | TK | 2014/4/19 | - | - |
| PLPgla26 | KP719367 | *Prionace glauca* | TK | 2014/4/19 | - | - |
| PLPgla27 | KP719368 | *Prionace glauca* | TK | 2014/4/19 | - | - |
| PLPgla28 | KP719369 | *Prionace glauca* | TK | 2014/4/19 | - | - |
| PLPgla29 | KP719370 | *Prionace glauca* | TK | 2014/4/19 | - | - |
| PLPgla30 | KP719371 | *Prionace glauca* | TK | 2014/4/19 | - | - |
| PLPgla31 | KP719372 | *Prionace glauca* | TK | 2014/4/19 | - | - |
| PLPgla32 | KP719373 | *Prionace glauca* | TK | 2014/4/19 | - | - |
| PLPgla33 | KP719374 | *Prionace glauca* | TK | 2014/4/19 | - | - |
| PLPgla34 | KP719375 | *Prionace glauca* | TK | 2014/4/19 | - | - |
| PLPgla35 | KP719376 | *Prionace glauca* | TK | 2014/4/19 | - | - |
| PLPgla36 | KP719377 | *Prionace glauca* | TK | 2014/4/19 | - | - |
| PLPgla37 | KP719378 | *Prionace glauca* | TK | 2014/4/19 | - | - |
| PLPgla38 | KP719379 | *Prionace glauca* | TK | 2014/4/19 | - | - |
| PLPgla39 | KP719380 | *Prionace glauca* | TK | 2014/4/19 | - | - |
| PLPgla40 | KP719381 | *Prionace glauca* | TK | 2014/4/19 | - | - |
| PLPgla41 | KP719382 | *Prionace glauca* | TK | 2014/4/19 | - | - |
| PLPgla42 | KP719383 | *Prionace glauca* | TK | 2014/4/19 | - | - |
| PLPgla43 | KP719384 | *Prionace glauca* | TK | 2014/4/19 | - | - |
| PLPgla44 | KP719385 | *Prionace glauca* | TK | 2014/4/19 | - | - |
| PLPgla45 | KP719386 | *Prionace glauca* | TK | 2014/4/19 | - | - |
| PLPgla46 | KP719387 | *Prionace glauca* | TK | 2014/4/19 | - | - |

Continued

| Code | Accession # | Species | Location | Date | Weight (g) | Shape |
| --- | --- | --- | --- | --- | --- | --- |
| PLPgla47 | KP719388 | *Prionace glauca* | TK | 2014/4/19 | - | - |
| PLPgla48 | KP719389 | *Prionace glauca* | TK | 2014/4/19 | - | - |
| PLPgla49 | KP719390 | *Prionace glauca* | TK | 2014/4/19 | - | - |
| PLPgla50 | KP719391 | *Prionace glauca* | TK | 2014/4/19 | - | - |
| PLPgla51 | KP719392 | *Prionace glauca* | TK | 2014/4/19 | - | - |
| PLPgla52 | KP719393 | *Prionace glauca* | TK | 2014/4/19 | - | - |
| PLPgla53 | KP719394 | *Prionace glauca* | TK | 2014/4/19 | - | - |
| PLPgla54 | KP719395 | *Prionace glauca* | TK | 2014/4/19 | - | - |
| PLPgla55 | KP719396 | *Prionace glauca* | TK | 2014/4/19 | - | - |
| PLPgla56 | KP719397 | *Prionace glauca* | TK | 2014/4/19 | - | - |
| PLPgla57 | KP719398 | *Prionace glauca* | TK | 2014/4/19 | - | - |
| PLPgla58 | KP719399 | *Prionace glauca* | TK | 2014/4/19 | - | - |
| PLPgla59 | KP719400 | *Prionace glauca* | TK | 2014/4/19 | - | - |
| PLPgla60 | KP719401 | *Prionace glauca* | TK | 2014/4/19 | - | - |
| PLPgla61 | KP719402 | *Prionace glauca* | TK | 2014/4/19 | - | - |
| PLPgla62 | KP719403 | *Prionace glauca* | TK | 2014/4/19 | - | - |
| PLPgla63 | KP719404 | *Prionace glauca* | TK | 2014/4/19 | - | - |
| PLPgla64 | KP719405 | *Prionace glauca* | TK | 2014/4/19 | - | - |
| PLPgla65 | KP719406 | *Prionace glauca* | TK | 2014/4/19 | - | - |
| PLPgla66 | KP719407 | *Prionace glauca* | TK | 2014/4/19 | - | - |
| PLPgla67 | KP719408 | *Prionace glauca* | TK | 2014/4/19 | - | - |
| PLPgla68 | KP719409 | *Prionace glauca* | TK | 2014/4/19 | - | - |
| PLPgla69 | KP719410 | *Prionace glauca* | TK | 2014/4/19 | - | - |
| PLPgla70 | KP719411 | *Prionace glauca* | TK | 2014/4/19 | - | - |
| PLPgla71 | KP719412 | *Prionace glauca* | TK | 2014/4/19 | - | - |
| PLPgla72 | KP719413 | *Prionace glauca* | NFA | 2013/6/4 | - | - |
| PLPgla73 | KP719414 | *Prionace glauca* | NFA | 2013/6/4 | - | - |
| PLPgla74 | KP719415 | *Prionace glauca* | NFA | 2013/6/4 | - | - |
| PLPgla75 | KP719416 | *Prionace glauca* | NFA | 2013/6/4 | - | - |
| PLPgla76 | KP719417 | *Prionace glauca* | NFA | 2013/6/4 | - | - |
| PLPgla77 | KP719418 | *Prionace glauca* | NFA | 2013/6/4 | - | - |
| PLPgla78 | KP719419 | *Prionace glauca* | NFA | 2013/6/4 | - | - |
| PLPgla79 | KP719420 | *Prionace glauca* | NFA | 2013/6/4 | - | - |
| PLPgla80 | KP719421 | *Prionace glauca* | NFA | 2013/6/4 | - | - |
| PLPgla81 | KP719422 | *Prionace glauca* | NFA | 2013/6/4 | - | - |
| PLPgla82 | KP719423 | *Prionace glauca* | NFA | 2013/6/4 | - | - |
| PLPgla83 | KP719424 | *Prionace glauca* | NFA | 2013/6/4 | - | - |
| PLPgla84 | KP719425 | *Prionace glauca* | NFA | 2013/6/4 | - | - |
| PLPgla85 | KP719426 | *Prionace glauca* | NFA | 2013/6/4 | - | - |
| PLPgla86 | KP719427 | *Prionace glauca* | NFA | 2013/6/4 | - | - |

Continued

| Code | Accession # | Species | Location | Date | Weight (g) | Shape |
| --- | --- | --- | --- | --- | --- | --- |
| PLPgla87 | KP719428 | *Prionace glauca* | XG | 2013/3/18 | - | - |
| PLPgla88 | KP719429 | *Prionace glauca* | XG | 2013/3/18 | - | - |
| PLPgla89 | KP719430 | *Prionace glauca* | XG | 2013/3/18 | - | - |
| PLPgla90 | KP719431 | *Prionace glauca* | XG | 2013/3/18 | - | - |
| PLPgla91 | KP719432 | *Prionace glauca* | XG | 2013/3/18 | - | - |
| PLPgla92 | KP719433 | *Prionace glauca* | XG | 2013/3/18 | - | - |
| PLPgla93 | KP719434 | *Prionace glauca* | XG | 2013/3/18 | - | - |
| PLPgla94 | KP719435 | *Prionace glauca* | XG | 2013/3/18 | - | - |
| PLPgla95 | KP719436 | *Prionace glauca* | TK | 2013/3/16 | - | - |
| PLPgla96 | KP719437 | *Prionace glauca* | TK | 2013/3/16 | - | - |
| PLPgla97 | KP719438 | *Prionace glauca* | TK | 2013/3/16 | - | - |
| PLPgla98 | KP719439 | *Prionace glauca* | TK | 2013/3/16 | - | - |
| PLPgla99 | KP719440 | *Prionace glauca* | TK | 2013/3/16 | - | - |
| PLPgla100 | KP719441 | *Prionace glauca* | TK | 2013/8/6 | - | - |
| PLPgla101 | KP719442 | *Prionace glauca* | TK | 2013/8/6 | - | - |
| PLPgla102 | KP719443 | *Prionace glauca* | TK | 2013/8/6 | - | - |
| PLPgla103 | KP719444 | *Prionace glauca* | TK | 2013/8/6 | - | - |
| PLPgla104 | KP719445 | *Prionace glauca* | TK | 2013/8/6 | - | - |
| PLPgla105 | KP719446 | *Prionace glauca* | TK | 2013/8/6 | - | - |
| PLPgla106 | KP719447 | *Prionace glauca* | TK | 2013/8/6 | - | - |
| PLPgla107 | KP719448 | *Prionace glauca* | TK | 2013/8/6 | - | - |
| PLPgla108 | KP719449 | *Prionace glauca* | TK | 2013/8/6 | - | - |
| PLPgla109 | KP719450 | *Prionace glauca* | TK | 2013/8/6 | - | - |
| PLSlew1 | KP719451 | *Sphyrna lewini* | XG | 2013/6/5 | - | - |
| PLSlew2 | KP719452 | *Sphyrna lewini* | XG | 2013/6/5 | - | - |
| PLSlew3 | KP719453 | *Sphyrna lewini* | XG | 2013/3/18 | - | - |
| PLSzyg1 | KP719454 | *Sphyrna zygaena* | XG | 2013/6/5 | - | - |
| PLSzyg2 | KP719455 | *Sphyrna zygaena* | XG | 2013/6/5 | - | - |
| PLSzyg3 | KP719456 | *Sphyrna zygaena* | XG | 2013/6/5 | - | - |
| PLSzyg4 | KP719457 | *Sphyrna zygaena* | XG | 2013/6/5 | - | - |
| PLSzyg5 | KP719458 | *Sphyrna zygaena* | XG | 2013/6/5 | - | - |
| FPIoxy1 | KP719459 | *Isurus oxyrinchus* | HG | 2012/10/7 | 4.53 | 2 |
| FPIoxy2 | KP719460 | *Isurus oxyrinchus* | HG | 2012/10/7 | 3.21 | 2 |
| FPIoxy3 | KP719461 | *Isurus oxyrinchus* | HG | 2012/10/7 | 4.41 | 2 |
| FPIoxy4 | KP719462 | *Isurus oxyrinchus* | WC | 2013/8/26 | 0.88 | 2 |
| FPIoxy5 | KP719463 | *Isurus oxyrinchus* | NGF | 2013/6/6 | 4.03 | 2 |
| FPApel1 | KP719464 | *Alopias pelagicus* | HG | 2012/10/7 | 19.89 | 1 |
| FPApel2 | KP719465 | *Alopias pelagicus* | WC | 2013/8/26 | 16.89 | 1 |
| FPApel3 | KP719466 | *Alopias pelagicus* | MY | 2013/3/30 | 18.65 | 2 |
| FPApel4 | KP719467 | *Alopias pelagicus* | MY | 2013/3/30 | 19.43 | 2 |

Continued

| Code | Accession # | Species | Location | Date | Weight (g) | Shape |
| --- | --- | --- | --- | --- | --- | --- |
| FPApel5 | KP719468 | *Alopias pelagicus* | MY | 2013/3/30 | 17.59 | 2 |
| FPAsup1 | KP719469 | *Alopias superciliosus* | WC | 2013/8/26 | 7.33 | 1 |
| FPAsup2 | KP719470 | *Alopias superciliosus* | MY | 2013/3/30 | 22.38 | 2 |
| FPCalt1 | KP719471 | *Carcharhinus altimus* | WC | 2013/8/26 | 0.58 | 3 |
| FPCamb1 | KP719472 | *Carcharhinus amboinensis* | JG | 2014/5/5 | 3.08 | 3 |
| FPCbre1 | KP719473 | *Carcharhinus brevipinna* | YL | 2014/4/6 | 3.31 | 3 |
| FPCbre2 | KP719474 | *Carcharhinus brevipinna* | YL | 2014/4/6 | 4.01 | 2 |
| FPCbre3 | KP719475 | *Carcharhinus brevipinna* | MY | 2013/3/30 | 9.06 | 2 |
| FPCcoa1 | KP719476 | *Carcharhinus coatesi* | LMY | 2013/9/9 | 3.96 | 2 |
| FPCcoa2 | KP719477 | *Carcharhinus coatesi* | LMY | 2013/9/9 | 4.28 | 2 |
| FPCcoa3 | KP719478 | *Carcharhinus coatesi* | LMY | 2013/9/9 | 3.69 | 2 |
| FPCcoa4 | KP719479 | *Carcharhinus coatesi* | LMY | 2013/9/9 | 5.48 | 2 |
| FPCcoa5 | KP719480 | *Carcharhinus coatesi* | LMY | 2013/9/9 | 2.93 | 2 |
| FPCcoa6 | KP719481 | *Carcharhinus coatesi* | LMY | 2013/9/9 | 4.99 | 2 |
| FPCcoa7 | KP719482 | *Carcharhinus coatesi* | LMY | 2013/9/9 | 4.09 | 2 |
| FPCcoa8 | KP719483 | *Carcharhinus coatesi* | LMY | 2013/9/9 | 6.14 | 2 |
| FPCcoa9 | KP719484 | *Carcharhinus coatesi* | LMY | 2013/9/9 | 2.01 | 3 |
| FPCcoa10 | KP719485 | *Carcharhinus coatesi* | LMY | 2013/9/9 | 1.83 | 3 |
| FPCcoa11 | KP719486 | *Carcharhinus coatesi* | LMY | 2013/9/9 | 0.89 | 3 |
| FPCcoa12 | KP719487 | *Carcharhinus coatesi* | YL | 2014/4/6 | 6.23 | 3 |
| FPCcoa13 | KP719488 | *Carcharhinus coatesi* | JG | 2014/5/5 | 3.11 | 2 |
| FPCcoa14 | KP719489 | *Carcharhinus coatesi* | JG | 2014/5/5 | 5.13 | 2 |
| FPCcoa15 | KP719490 | *Carcharhinus coatesi* | JG | 2014/5/5 | 4.84 | 2 |
| FPCcoa16 | KP719491 | *Carcharhinus coatesi* | JG | 2014/5/5 | 4.95 | 2 |
| FPCcoa17 | KP719492 | *Carcharhinus coatesi* | JG | 2014/5/5 | 3.68 | 2 |
| FPCcoa18 | KP719493 | *Carcharhinus coatesi* | JG | 2014/5/5 | 3.85 | 2 |
| FPCcoa19 | KP719494 | *Carcharhinus coatesi* | JG | 2014/5/5 | 2.07 | 2 |
| FPCcoa20 | KP719495 | *Carcharhinus coatesi* | JG | 2014/5/5 | 2.28 | 2 |
| FPCcoa21 | KP719496 | *Carcharhinus coatesi* | JG | 2014/5/5 | 2.64 | 2 |
| FPCcoa22 | KP719497 | *Carcharhinus coatesi* | JG | 2014/5/5 | 4.78 | 3 |
| FPCcoa23 | KP719498 | *Carcharhinus coatesi* | JG | 2014/5/5 | 3.83 | 3 |
| FPCcoa24 | KP719499 | *Carcharhinus coatesi* | JG | 2014/5/5 | 2.69 | 3 |
| FPCcoa25 | KP719500 | *Carcharhinus coatesi* | JG | 2014/5/5 | 2.3 | 3 |
| FPCcoa26 | KP719501 | *Carcharhinus coatesi* | JG | 2014/5/5 | 3.52 | 3 |
| FPCcoa27 | KP719502 | *Carcharhinus coatesi* | JG | 2014/5/5 | 2.55 | 3 |
| FPCcoa28 | KP719503 | *Carcharhinus coatesi* | NGF | 2013/7/25 | - | - |
| FPCcoa29 | KP719504 | *Carcharhinus coatesi* | NGF | 2013/7/25 | - | - |
| FPCcoa30 | KP719505 | *Carcharhinus coatesi* | NGF | 2013/7/25 | - | - |
| FPCcoa31 | KP719506 | *Carcharhinus coatesi* | NGF | 2013/7/25 | - | - |
| FPCcoa32 | KP719507 | *Carcharhinus coatesi* | NGF | 2013/7/25 | - | - |

Continued

| Code | Accession # | Species | Location | Date | Weight (g) | Shape |
| --- | --- | --- | --- | --- | --- | --- |
| FPCcoa33 | KP719508 | *Carcharhinus coatesi* | NGF | 2013/7/25 | - | - |
| FPCcoa34 | KP719509 | *Carcharhinus coatesi* | NGF | 2013/7/25 | - | - |
| FPCcoa35 | KP719510 | *Carcharhinus coatesi* | NGF | 2013/7/25 | - | - |
| FPCcoa36 | KP719511 | *Carcharhinus coatesi* | NGF | 2013/7/25 | - | - |
| FPCcoa37 | KP719512 | *Carcharhinus coatesi* | NGF | 2013/7/25 | - | - |
| FPCfal1 | KP719513 | *Carcharhinus falciformis* | HG | 2012/10/7 | 3.33 | 2 |
| FPCfal2 | KP719514 | *Carcharhinus falciformis* | HG | 2012/10/7 | 4.1 | 2 |
| FPCfal3 | KP719515 | *Carcharhinus falciformis* | HG | 2012/10/7 | 4.44 | 2 |
| FPCfal4 | KP719516 | *Carcharhinus falciformis* | HG | 2012/10/7 | 3.25 | 2 |
| FPCfal5 | KP719517 | *Carcharhinus falciformis* | HG | 2012/10/7 | 2.88 | 2 |
| FPCfal6 | KP719518 | *Carcharhinus falciformis* | NGF | 2013/9/9 | 8.42 | 2 |
| FPCfal7 | KP719519 | *Carcharhinus falciformis* | NGF | 2013/9/9 | 14.86 | 2 |
| FPCfal8 | KP719520 | *Carcharhinus falciformis* | NGF | 2013/9/9 | 11.91 | 2 |
| FPCfal9 | KP719521 | *Carcharhinus falciformis* | NGF | 2013/9/9 | 10.72 | 2 |
| FPCfal10 | KP719522 | *Carcharhinus falciformis* | NGF | 2013/9/9 | 8.03 | 2 |
| FPCfal11 | KP719523 | *Carcharhinus falciformis* | NGF | 2013/9/9 | 4.59 | 2 |
| FPCfal12 | KP719524 | *Carcharhinus falciformis* | NGF | 2013/9/9 | 7.76 | 2 |
| FPCfal13 | KP719525 | *Carcharhinus falciformis* | NGF | 2013/9/9 | 9.43 | 2 |
| FPCfal14 | KP719526 | *Carcharhinus falciformis* | NGF | 2013/9/9 | 6.17 | 2 |
| FPCfal15 | KP719527 | *Carcharhinus falciformis* | WC | 2013/8/26 | 0.39 | 3 |
| FPCfal16 | KP719528 | *Carcharhinus falciformis* | WC | 2013/8/26 | 0.43 | 3 |
| FPCfal17 | KP719529 | *Carcharhinus falciformis* | WC | 2013/8/26 | 6.23 | 1 |
| FPCfal18 | KP719530 | *Carcharhinus falciformis* | WC | 2013/8/26 | 4.06 | 1 |
| FPCfal19 | KP719531 | *Carcharhinus falciformis* | WC | 2013/8/26 | 3.25 | 1 |
| FPCfal20 | KP719532 | *Carcharhinus falciformis* | WC | 2013/8/26 | 3.69 | 1 |
| FPCfal21 | KP719533 | *Carcharhinus falciformis* | LMY | 2013/9/9 | 23.42 | 2 |
| FPCfal22 | KP719534 | *Carcharhinus falciformis* | LMY | 2013/9/9 | 19.79 | 1 |
| FPCfal23 | KP719535 | *Carcharhinus falciformis* | LMY | 2013/9/9 | 19.12 | 2 |
| FPCfal24 | KP719536 | *Carcharhinus falciformis* | LMY | 2013/9/9 | 15.41 | 2 |
| FPCfal25 | KP719537 | *Carcharhinus falciformis* | LMY | 2013/9/9 | 17.09 | 2 |
| FPCfal26 | KP719538 | *Carcharhinus falciformis* | LMY | 2013/9/9 | 13.77 | 2 |
| FPCfal27 | KP719539 | *Carcharhinus falciformis* | SY | 2013/9/21 | 4.96 | 3 |
| FPCfal28 | KP719540 | *Carcharhinus falciformis* | SY | 2013/9/21 | 6.91 | 2 |
| FPCfal29 | KP719541 | *Carcharhinus falciformis* | SY | 2013/9/21 | 7.1 | 3 |
| FPCfal30 | KP719542 | *Carcharhinus falciformis* | MY | 2013/3/30 | 22.38 | 2 |
| FPCfal31 | KP719543 | *Carcharhinus falciformis* | MY | 2013/3/30 | 14.17 | 1 |
| FPCfal32 | KP719544 | *Carcharhinus falciformis* | MY | 2013/3/30 | 3.04 | 3 |
| FPCfal33 | KP719545 | *Carcharhinus falciformis* | MY | 2013/3/30 | 2.36 | 3 |
| FPCfal34 | KP719546 | *Carcharhinus falciformis* | MY | 2013/3/30 | 2.04 | 3 |
| FPCfal35 | KP719547 | *Carcharhinus falciformis* | MY | 2013/3/30 | 3.2 | 3 |

Continued

| Code | Accession # | Species | Location | Date | Weight (g) | Shape |
| --- | --- | --- | --- | --- | --- | --- |
| FPCfal36 | KP719548 | *Carcharhinus falciformis* | MY | 2013/3/30 | 1.74 | 2 |
| FPCfal37 | KP719549 | *Carcharhinus falciformis* | MY | 2013/3/30 | 2.46 | 3 |
| FPCfal38 | KP719550 | *Carcharhinus falciformis* | MY | 2013/3/30 | 1.29 | 3 |
| FPCfal39 | KP719551 | *Carcharhinus falciformis* | MY | 2013/3/30 | 3.4 | 2 |
| FPCfal40 | KP719552 | *Carcharhinus falciformis* | MY | 2013/3/30 | 2.62 | 3 |
| FPCfal41 | KP719553 | *Carcharhinus falciformis* | MY | 2013/3/30 | 1.45 | 3 |
| FPCfal42 | KP719554 | *Carcharhinus falciformis* | NGF | 2013/7/25 | - | - |
| FPCfal43 | KP719555 | *Carcharhinus falciformis* | NGF | 2013/7/25 | - | - |
| FPClim1 | KP719556 | *Carcharhinus limbatus* | WC | 2013/8/26 | 3.87 | 1 |
| FPClim2 | KP719557 | *Carcharhinus limbatus* | JG | 2014/5/5 | 3.46 | 2 |
| FPClim3 | KP719558 | *Carcharhinus limbatus* | JG | 2014/5/5 | 3.06 | 2 |
| FPClim4 | KP719559 | *Carcharhinus limbatus* | JG | 2014/5/5 | 5.33 | 2 |
| FPClim5 | KP719560 | *Carcharhinus limbatus* | JG | 2014/5/5 | 3.02 | 2 |
| FPClon1 | KP719561 | *Carcharhinus longimanus* | HG | 2012/10/7 | 2.97 | 2 |
| FPClon2 | KP719562 | *Carcharhinus longimanus* | HG | 2012/10/7 | 3.87 | 2 |
| FPClon3 | KP719563 | *Carcharhinus longimanus* | NGF | 2013/6/6 | 1.34 | 2 |
| FPClon4 | KP719564 | *Carcharhinus longimanus* | NGF | 2013/7/25 | - | - |
| FPCmac1 | KP719565 | *Carcharhinus macloti* | YL | 2014/4/6 | 2.9 | 2 |
| FPCmac2 | KP719566 | *Carcharhinus macloti* | YL | 2014/4/6 | 2.58 | 2 |
| FPCmac3 | KP719567 | *Carcharhinus macloti* | YL | 2014/4/6 | 3.03 | 3 |
| FPCmac4 | KP719568 | *Carcharhinus macloti* | YL | 2014/4/6 | 4.16 | 3 |
| FPCmac5 | KP719569 | *Carcharhinus macloti* | YL | 2014/4/6 | 3.94 | 3 |
| FPCmac6 | KP719570 | *Carcharhinus macloti* | YL | 2014/4/6 | 4.37 | 2 |
| FPCmac7 | KP719571 | *Carcharhinus macloti* | YL | 2014/4/6 | 4.42 | 2 |
| FPCmac8 | KP719572 | *Carcharhinus macloti* | YL | 2014/4/6 | 5.5 | 2 |
| FPCmac9 | KP719573 | *Carcharhinus macloti* | YL | 2014/4/6 | 7.21 | 3 |
| FPCmac10 | KP719574 | *Carcharhinus macloti* | YL | 2014/4/6 | 2.71 | 2 |
| FPCmac11 | KP719575 | *Carcharhinus macloti* | YL | 2014/4/6 | 2.02 | 3 |
| FPCmac12 | KP719576 | *Carcharhinus macloti* | YL | 2014/4/6 | 3.52 | 3 |
| FPCmac13 | KP719577 | *Carcharhinus macloti* | YL | 2014/4/6 | 2.84 | 3 |
| FPCmac14 | KP719578 | *Carcharhinus macloti* | YL | 2014/4/6 | 2.58 | 2 |
| FPCmac15 | KP719579 | *Carcharhinus macloti* | YL | 2014/4/6 | 5.08 | 2 |
| FPCmac16 | KP719580 | *Carcharhinus macloti* | YL | 2014/4/6 | 3.53 | 3 |
| FPCmac17 | KP719581 | *Carcharhinus macloti* | YL | 2014/4/6 | 2.9 | 2 |
| FPCmac18 | KP719582 | *Carcharhinus macloti* | YL | 2014/4/6 | 3.85 | 3 |
| FPCmac19 | KP719583 | *Carcharhinus macloti* | YL | 2014/4/6 | 4.06 | 3 |
| FPCmac20 | KP719584 | *Carcharhinus macloti* | YL | 2014/4/6 | 1.85 | 3 |
| FPCmac21 | KP719585 | *Carcharhinus macloti* | YL | 2014/4/6 | 3.53 | 3 |
| FPCmac22 | KP719586 | *Carcharhinus macloti* | JG | 2014/5/5 | 2.24 | 2 |
| FPCmac23 | KP719587 | *Carcharhinus macloti* | JG | 2014/5/5 | 3.56 | 2 |

Continued

| Code | Accession # | Species | Location | Date | Weight (g) | Shape |
| --- | --- | --- | --- | --- | --- | --- |
| FPCmac24 | KP719588 | *Carcharhinus macloti* | JG | 2014/5/5 | 5.66 | 2 |
| FPCmac25 | KP719589 | *Carcharhinus macloti* | JG | 2014/5/5 | 3.1 | 3 |
| FPCmac26 | KP719590 | *Carcharhinus macloti* | JG | 2014/5/5 | 3.54 | 2 |
| FPCmac27 | KP719591 | *Carcharhinus macloti* | JG | 2014/5/5 | 3.12 | 2 |
| FPCmac28 | KP719592 | *Carcharhinus macloti* | JG | 2014/5/5 | 2.74 | 2 |
| FPCmac29 | KP719593 | *Carcharhinus macloti* | NGF | 2013/7/25 | - | - |
| FPCmac30 | KP719594 | *Carcharhinus macloti* | NGF | 2013/7/25 | - | - |
| FPCmac31 | KP719595 | *Carcharhinus macloti* | NGF | 2013/7/25 | - | - |
| FPCmac32 | KP719596 | *Carcharhinus macloti* | NGF | 2013/7/25 | - | - |
| FPCsea1 | KP719597 | *Carcharhinus sealei* | JG | 2014/5/5 | 3.97 | 2 |
| FPCsea2 | KP719598 | *Carcharhinus sealei* | JG | 2014/5/5 | 5.42 | 2 |
| FPCsea3 | KP719599 | *Carcharhinus sealei* | JG | 2014/5/5 | 3.26 | 3 |
| FPCsea4 | KP719600 | *Carcharhinus sealei* | JG | 2014/5/5 | 3.48 | 2 |
| FPCsea5 | KP719601 | *Carcharhinus sealei* | JG | 2014/5/5 | 2.71 | 2 |
| FPCsea6 | KP719602 | *Carcharhinus sealei* | SK | 2014/6/13 | 1.02 | 3 |
| FPCsor1 | KP719603 | *Carcharhinus sorrah* | LMY | 2013/9/9 | 1.62 | 3 |
| FPCsor2 | KP719604 | *Carcharhinus sorrah* | LMY | 2013/9/9 | 3.69 | 2 |
| FPCsor3 | KP719605 | *Carcharhinus sorrah* | YL | 2014/4/6 | 7.8 | 2 |
| FPCsor4 | KP719606 | *Carcharhinus sorrah* | JG | 2014/5/5 | 5.28 | 2 |
| FPCsor5 | KP719607 | *Carcharhinus sorrah* | JG | 2014/5/5 | 3.49 | 3 |
| FPCsor6 | KP719608 | *Carcharhinus sorrah* | SK | 2014/6/13 | 1.31 | 1 |
| FPCsor7 | KP719609 | *Carcharhinus sorrah* | NGF | 2013/7/25 | - | - |
| FPCsor8 | KP719610 | *Carcharhinus sorrah* | NGF | 2013/7/25 | - | - |
| FPCsor9 | KP719611 | *Carcharhinus sorrah* | YL | 2014/4/6 | 3.76 | 2 |
| FPCtju1 | KP719612 | *Carcharhinus tjutjot* | JG | 2014/5/5 | 4.53 | 2 |
| FPGcuv1 | KP719613 | *Galeocerdo cuvier* | HG | 2012/10/7 | 3.55 | 2 |
| FPGcuv2 | KP719614 | *Galeocerdo cuvier* | MY | 2013/3/30 | 7.07 | 2 |
| FPHaus1 | KP719615 | *Hemigaleus australiensis* | LMY | 2013/9/9 | 5.26 | 2 |
| FPHaus2 | KP719616 | *Hemigaleus australiensis* | LMY | 2013/9/9 | 5.58 | 2 |
| FPHaus3 | KP719617 | *Hemigaleus australiensis* | LMY | 2013/9/9 | 4.75 | 3 |
| FPHaus4 | KP719618 | *Hemigaleus australiensis* | LMY | 2013/9/9 | 8.69 | 2 |
| FPHaus5 | KP719619 | *Hemigaleus australiensis* | LMY | 2013/9/9 | 4.17 | 2 |
| FPHaus6 | KP719620 | *Hemigaleus australiensis* | LMY | 2013/9/9 | 3.63 | 2 |
| FPHaus7 | KP719621 | *Hemigaleus australiensis* | LMY | 2013/9/9 | 1.99 | 2 |
| FPHaus8 | KP719622 | *Hemigaleus australiensis* | YL | 2014/4/6 | 3.29 | 3 |
| FPHaus9 | KP719623 | *Hemigaleus australiensis* | YL | 2014/4/6 | 2.83 | 2 |
| FPHaus10 | KP719624 | *Hemigaleus australiensis* | YL | 2014/4/6 | 2.49 | 3 |
| FPHaus11 | KP719625 | *Hemigaleus australiensis* | YL | 2014/4/6 | 4.87 | 3 |
| FPHaus12 | KP719626 | *Hemigaleus australiensis* | YL | 2014/4/6 | 4.09 | 3 |
| FPHaus13 | KP719627 | *Hemigaleus australiensis* | YL | 2014/4/6 | 3.07 | 2 |

Continued

| Code | Accession # | Species | Location | Date | Weight (g) | Shape |
| --- | --- | --- | --- | --- | --- | --- |
| FPHaus14 | KP719628 | *Hemigaleus australiensis* | YL | 2014/4/6 | 4.55 | 3 |
| FPHaus15 | KP719629 | *Hemigaleus australiensis* | YL | 2014/4/6 | 2.8 | 3 |
| FPHaus16 | KP719630 | *Hemigaleus australiensis* | YL | 2014/4/6 | 5.33 | 3 |
| FPHaus17 | KP719631 | *Hemigaleus australiensis* | YL | 2014/4/6 | 4.5 | 3 |
| FPHaus18 | KP719632 | *Hemigaleus australiensis* | YL | 2014/4/6 | 0.76 | 3 |
| FPLnas1 | KP719633 | *Lamna nasus* | WC | 2013/8/26 | 6.95 | 1 |
| FPLnas2 | KP719634 | *Lamna nasus* | WC | 2013/8/26 | 3.34 | 1 |
| FPLnas3 | KP719635 | *Lamna nasus* | MY | 2013/3/30 | 22.82 | 1 |
| FPLnas4 | KP719636 | *Lamna nasus* | MY | 2013/3/30 | 12.68 | 1 |
| FPLmac1 | KP719637 | *Loxodon macrorhinus* | YL | 2014/4/6 | 3.48 | 3 |
| FPPgla1 | KP719638 | *Prionace glauca* | HG | 2012/10/7 | 4.76 | 2 |
| FPPgla2 | KP719639 | *Prionace glauca* | HG | 2012/10/7 | 5.77 | 2 |
| FPPgla3 | KP719640 | *Prionace glauca* | HG | 2012/10/7 | 5.96 | 2 |
| FPPgla4 | KP719641 | *Prionace glauca* | HG | 2012/10/7 | 6.1 | 2 |
| FPPgla5 | KP719642 | *Prionace glauca* | HG | 2012/10/7 | 2.39 | 2 |
| FPPgla6 | KP719643 | *Prionace glauca* | HG | 2012/10/7 | 2.74 | 2 |
| FPPgla7 | KP719644 | *Prionace glauca* | HG | 2012/10/7 | 4.94 | 2 |
| FPPgla8 | KP719645 | *Prionace glauca* | HG | 2012/10/7 | 4.89 | 2 |
| FPPgla9 | KP719646 | *Prionace glauca* | HG | 2012/10/7 | 2.84 | 2 |
| FPPgla10 | KP719647 | *Prionace glauca* | HG | 2012/10/7 | 4.89 | 2 |
| FPPgla11 | KP719648 | *Prionace glauca* | HG | 2012/10/7 | 4.12 | 2 |
| FPPgla12 | KP719649 | *Prionace glauca* | HG | 2012/10/7 | 2.79 | 3 |
| FPPgla13 | KP719650 | *Prionace glauca* | HG | 2012/10/7 | 3.38 | 2 |
| FPPgla14 | KP719651 | *Prionace glauca* | HG | 2012/10/7 | 3.95 | 2 |
| FPPgla15 | KP719652 | *Prionace glauca* | HG | 2012/10/7 | 4.56 | 2 |
| FPPgla16 | KP719653 | *Prionace glauca* | HG | 2012/10/7 | 3.05 | 2 |
| FPPgla17 | KP719654 | *Prionace glauca* | HG | 2012/10/7 | 4.41 | 2 |
| FPPgla18 | KP719655 | *Prionace glauca* | HG | 2012/10/7 | 4.89 | 2 |
| FPPgla19 | KP719656 | *Prionace glauca* | HG | 2012/10/7 | 4.31 | 2 |
| FPPgla20 | KP719657 | *Prionace glauca* | HG | 2012/10/7 | 4.55 | 2 |
| FPPgla21 | KP719658 | *Prionace glauca* | NGF | 2013/9/9 | 37.56 | 2 |
| FPPgla22 | KP719659 | *Prionace glauca* | NGF | 2013/9/9 | 34.16 | 2 |
| FPPgla23 | KP719660 | *Prionace glauca* | NGF | 2013/9/9 | 48.53 | 1 |
| FPPgla24 | KP719661 | *Prionace glauca* | NGF | 2013/9/9 | 50.74 | 1 |
| FPPgla25 | KP719662 | *Prionace glauca* | NGF | 2013/9/9 | 45.31 | 1 |
| FPPgla26 | KP719663 | *Prionace glauca* | NGF | 2013/9/9 | 27.42 | 1 |
| FPPgla27 | KP719664 | *Prionace glauca* | NGF | 2013/9/9 | 24.53 | 2 |
| FPPgla28 | KP719665 | *Prionace glauca* | NGF | 2013/9/9 | 17.99 | 2 |
| FPPgla29 | KP719666 | *Prionace glauca* | NGF | 2013/9/9 | 12 | 2 |
| FPPgla30 | KP719667 | *Prionace glauca* | NGF | 2013/9/9 | 10.15 | 1 |

Continued

| Code | Accession # | Species | Location | Date | Weight (g) | Shape |
| --- | --- | --- | --- | --- | --- | --- |
| FPPgla31 | KP719668 | *Prionace glauca* | NGF | 2013/9/9 | 10.23 | 1 |
| FPPgla32 | KP719669 | *Prionace glauca* | NGF | 2013/9/9 | 9.33 | 3 |
| FPPgla33 | KP719670 | *Prionace glauca* | NGF | 2013/9/9 | 8.76 | 2 |
| FPPgla34 | KP719671 | *Prionace glauca* | NGF | 2013/9/9 | 9.96 | 2 |
| FPPgla35 | KP719672 | *Prionace glauca* | NGF | 2013/9/9 | 8.56 | 2 |
| FPPgla36 | KP719673 | *Prionace glauca* | NGF | 2013/9/9 | 1.59 | 1 |
| FPPgla37 | KP719674 | *Prionace glauca* | NGF | 2013/9/9 | 2.46 | 2 |
| FPPgla38 | KP719675 | *Prionace glauca* | NGF | 2013/9/9 | 26.16 | 2 |
| FPPgla39 | KP719676 | *Prionace glauca* | NGF | 2013/9/9 | 2.58 | 3 |
| FPPgla40 | KP719677 | *Prionace glauca* | WC | 2013/8/26 | 0.93 | 2 |
| FPPgla41 | KP719678 | *Prionace glauca* | WC | 2013/8/26 | 0.61 | 3 |
| FPPgla42 | KP719679 | *Prionace glauca* | WC | 2013/8/26 | 0.82 | 3 |
| FPPgla43 | KP719680 | *Prionace glauca* | WC | 2013/8/26 | 1.58 | 1 |
| FPPgla44 | KP719681 | *Prionace glauca* | WC | 2013/8/26 | 1.54 | 3 |
| FPPgla45 | KP719682 | *Prionace glauca* | WC | 2013/8/26 | 1.19 | 2 |
| FPPgla46 | KP719683 | *Prionace glauca* | WC | 2013/8/26 | 0.7 | 3 |
| FPPgla47 | KP719684 | *Prionace glauca* | WC | 2013/8/26 | 14.29 | 2 |
| FPPgla48 | KP719685 | *Prionace glauca* | DY | 2014/6/9 | 15.36 | 1 |
| FPPgla49 | KP719686 | *Prionace glauca* | DY | 2014/6/9 | 3.52 | 3 |
| FPPgla50 | KP719687 | *Prionace glauca* | DY | 2014/6/9 | 7.64 | 1 |
| FPPgla51 | KP719688 | *Prionace glauca* | DY | 2014/6/9 | 22.96 | 1 |
| FPPgla52 | KP719689 | *Prionace glauca* | DY | 2014/6/9 | 7.95 | 3 |
| FPPgla53 | KP719690 | *Prionace glauca* | DY | 2014/6/9 | 8.24 | 1 |
| FPPgla54 | KP719691 | *Prionace glauca* | DY | 2014/6/9 | 13.75 | 1 |
| FPPgla55 | KP719692 | *Prionace glauca* | DY | 2014/6/9 | 12.74 | 1 |
| FPPgla56 | KP719693 | *Prionace glauca* | DY | 2014/6/9 | 10.81 | 2 |
| FPPgla57 | KP719694 | *Prionace glauca* | DY | 2014/6/9 | 11.45 | 3 |
| FPPgla58 | KP719695 | *Prionace glauca* | DY | 2014/6/9 | 9.51 | 2 |
| FPPgla59 | KP719696 | *Prionace glauca* | SY | 2013/9/21 | 5.51 | 3 |
| FPPgla60 | KP719697 | *Prionace glauca* | SY | 2013/9/21 | 3.87 | 3 |
| FPPgla61 | KP719698 | *Prionace glauca* | SY | 2013/9/21 | 4.68 | 3 |
| FPPgla62 | KP719699 | *Prionace glauca* | SY | 2013/9/21 | 4.65 | 3 |
| FPPgla63 | KP719700 | *Prionace glauca* | SY | 2013/9/21 | 3.48 | 3 |
| FPPgla64 | KP719701 | *Prionace glauca* | SY | 2013/9/21 | 3.36 | 3 |
| FPPgla65 | KP719702 | *Prionace glauca* | SY | 2013/9/21 | 2.46 | 3 |
| FPPgla66 | KP719703 | *Prionace glauca* | SY | 2013/9/21 | 29.04 | 1 |
| FPPgla67 | KP719704 | *Prionace glauca* | SY | 2013/9/21 | 16.31 | 1 |
| FPPgla68 | KP719705 | *Prionace glauca* | SY | 2013/9/21 | 14.17 | 1 |
| FPPgla69 | KP719706 | *Prionace glauca* | NGF | 2013/6/6 | 4.22 | 2 |
| FPPgla70 | KP719707 | *Prionace glauca* | NGF | 2013/6/6 | 4.03 | 2 |

Continued

| Code | Accession # | Species | Location | Date | Weight (g) | Shape |
| --- | --- | --- | --- | --- | --- | --- |
| FPPgla71 | KP719708 | *Prionace glauca* | NGF | 2013/6/6 | 3.42 | 2 |
| FPPgla72 | KP719709 | *Prionace glauca* | NGF | 2013/6/6 | 2.86 | 2 |
| FPPgla73 | KP719710 | *Prionace glauca* | NGF | 2013/6/6 | 2.47 | 2 |
| FPPgla74 | KP719711 | *Prionace glauca* | NGF | 2013/6/6 | 3.85 | 2 |
| FPPgla75 | KP719712 | *Prionace glauca* | NGF | 2013/6/6 | 2.26 | 3 |
| FPPgla76 | KP719713 | *Prionace glauca* | NGF | 2013/6/6 | 2.32 | 2 |
| FPPgla77 | KP719714 | *Prionace glauca* | NGF | 2013/6/6 | 3.2 | 2 |
| FPPgla78 | KP719715 | *Prionace glauca* | NGF | 2013/6/6 | 2.91 | 2 |
| FPPgla79 | KP719716 | *Prionace glauca* | NGF | 2013/6/6 | 2.05 | 2 |
| FPPgla80 | KP719717 | *Prionace glauca* | NGF | 2013/6/6 | 2.21 | 2 |
| FPPgla81 | KP719718 | *Prionace glauca* | NGF | 2013/6/6 | 2.36 | 2 |
| FPPgla82 | KP719719 | *Prionace glauca* | NGF | 2013/6/6 | 3.09 | 2 |
| FPPgla83 | KP719720 | *Prionace glauca* | NGF | 2013/6/6 | 2.82 | 2 |
| FPPgla84 | KP719721 | *Prionace glauca* | NGF | 2013/6/6 | 3.4 | 2 |
| FPPgla85 | KP719722 | *Prionace glauca* | NGF | 2013/6/6 | 1.53 | 3 |
| FPPgla86 | KP719723 | *Prionace glauca* | NGF | 2013/6/6 | 2.31 | 2 |
| FPPgla87 | KP719724 | *Prionace glauca* | NGF | 2013/6/6 | 1.56 | 2 |
| FPPgla88 | KP719725 | *Prionace glauca* | NGF | 2013/6/6 | 2.64 | 2 |
| FPPgla89 | KP719726 | *Prionace glauca* | NGF | 2013/6/6 | 2.18 | 2 |
| FPPgla90 | KP719727 | *Prionace glauca* | NGF | 2013/6/6 | 2.18 | 2 |
| FPPgla91 | KP719728 | *Prionace glauca* | NGF | 2013/6/6 | 2.12 | 2 |
| FPPgla92 | KP719729 | *Prionace glauca* | NGF | 2013/6/6 | 2.49 | 2 |
| FPPgla93 | KP719730 | *Prionace glauca* | MY | 2013/3/30 | 2.61 | 3 |
| FPPgla94 | KP719731 | *Prionace glauca* | SK | 2014/6/13 | 49.51 | 1 |
| FPPgla95 | KP719732 | *Prionace glauca* | SK | 2014/6/13 | 30.46 | 1 |
| FPPgla96 | KP719733 | *Prionace glauca* | SK | 2014/6/13 | 38.48 | 1 |
| FPPgla97 | KP719734 | *Prionace glauca* | NGF | 2013/7/25 | - | - |
| FPPgla98 | KP719735 | *Prionace glauca* | NGF | 2013/7/25 | - | - |
| FPPgla99 | KP719736 | *Prionace glauca* | NGF | 2013/7/25 | - | - |
| FPPgla100 | KP719737 | *Prionace glauca* | NGF | 2013/7/25 | - | - |
| FPPgla101 | KP719738 | *Prionace glauca* | NGF | 2013/7/25 | - | - |
| FPPgla102 | KP719739 | *Prionace glauca* | NGF | 2013/7/25 | - | - |
| FPPgla103 | KP719740 | *Prionace glauca* | NGF | 2013/7/25 | - | - |
| FPPgla104 | KP719741 | *Prionace glauca* | NGF | 2013/7/25 | - | - |
| FPPgla105 | KP719742 | *Prionace glauca* | NGF | 2013/7/25 | - | - |
| FPPgla106 | KP719743 | *Prionace glauca* | NGF | 2013/7/25 | - | - |
| FPRacu1 | KP719744 | *Rhizoprionodon acutus* | WC | 2013/8/26 | 0.43 | 3 |
| FPRacu2 | KP719745 | *Rhizoprionodon acutus* | YL | 2014/4/6 | 5.46 | 2 |
| FPRacu3 | KP719746 | *Rhizoprionodon acutus* | JG | 2014/5/5 | 3.03 | 2 |
| FPRacu4 | KP719747 | *Rhizoprionodon acutus* | JG | 2014/5/5 | 3.29 | 2 |

Continued

| Code | Accession # | Species | Location | Date | Weight (g) | Shape |
| --- | --- | --- | --- | --- | --- | --- |
| FPRacu5 | KP719748 | *Rhizoprionodon acutus* | JG | 2014/5/5 | 4.73 | 3 |
| FPRacu6 | KP719749 | *Rhizoprionodon acutus* | NGF | 2013/7/25 | - | - |
| FPRtay1 | KP719750 | *Rhizoprionodon taylori* | YL | 2014/4/6 | 2.76 | 3 |
| FPRtay2 | KP719751 | *Rhizoprionodon taylori* | YL | 2014/4/6 | 2.54 | 3 |
| FPRtay3 | KP719752 | *Rhizoprionodon taylori* | YL | 2014/4/6 | 3.3 | 2 |
| FPRaus1 | KP719753 | *Rhynchobatus australiae* | WC | 2013/8/26 | 0.7 | 3 |
| FPSlew1 | KP719754 | *Sphyrna lewini* | HG | 2012/10/7 | 21.2 | 1 |
| FPSlew2 | KP719755 | *Sphyrna lewini* | NGF | 2013/9/9 | 2.95 | 2 |
| FPSlew3 | KP719756 | *Sphyrna lewini* | WC | 2013/8/26 | 0.87 | 3 |
| FPSlew4 | KP719757 | *Sphyrna lewini* | WC | 2013/8/26 | 0.84 | 3 |
| FPSlew5 | KP719758 | *Sphyrna lewini* | WC | 2013/8/26 | 0.7 | 3 |
| FPSlew6 | KP719759 | *Sphyrna lewini* | WC | 2013/8/26 | 0.14 | 3 |
| FPSlew7 | KP719760 | *Sphyrna lewini* | LMY | 2013/9/9 | 6.84 | 2 |
| FPSlew8 | KP719761 | *Sphyrna lewini* | LMY | 2013/9/9 | 5.61 | 2 |
| FPSlew9 | KP719762 | *Sphyrna lewini* | LMY | 2013/9/9 | 1.75 | 3 |
| FPSlew10 | KP719763 | *Sphyrna lewini* | YL | 2014/4/6 | 3.43 | 3 |
| FPSlew11 | KP719764 | *Sphyrna lewini* | YL | 2014/4/6 | 1.77 | 3 |
| FPSlew12 | KP719765 | *Sphyrna lewini* | YL | 2014/4/6 | 1.8 | 3 |
| FPSlew13 | KP719766 | *Sphyrna lewini* | YL | 2014/4/6 | 4.2 | 2 |
| FPSlew14 | KP719767 | *Sphyrna lewini* | JG | 2014/5/5 | 5.47 | 2 |
| FPSlew15 | KP719768 | *Sphyrna lewini* | JG | 2014/5/5 | 3.64 | 2 |
| FPSlew16 | KP719769 | *Sphyrna lewini* | JG | 2014/5/5 | 4.44 | 2 |
| FPSlew17 | KP719770 | *Sphyrna lewini* | JG | 2014/5/5 | 4.46 | 3 |
| FPSlew18 | KP719771 | *Sphyrna lewini* | MY | 2013/3/30 | 25.9 | 2 |
| FPSlew19 | KP719772 | *Sphyrna lewini* | MY | 2013/3/30 | 26.53 | 2 |
| FPSlew20 | KP719773 | *Sphyrna lewini* | NGF | 2013/7/25 | - | - |
| FPStib1 | KP719774 | *Sphyrna tiburo* | WC | 2013/8/26 | 0.61 | 3 |
| CDApel1 | KP719775 | *Alopias pelagicus* | KC | 2014/3/12 | - | - |
| CDApel2 | KP719776 | *Alopias pelagicus* | KC | 2014/3/12 | - | - |
| CDApel3 | KP719777 | *Alopias pelagicus* | KC | 2014/3/12 | - | - |
| CDApel4 | KP719778 | *Alopias pelagicus* | KC | 2014/3/12 | - | - |
| CDApel5 | KP719779 | *Alopias pelagicus* | KC | 2013/12/4 | - | - |
| CDApel6 | KP719780 | *Alopias pelagicus* | KC | 2013/12/4 | - | - |
| CDApel7 | KP719781 | *Alopias pelagicus* | KC | 2014/5/22 | - | - |
| CDApel8 | KP719782 | *Alopias pelagicus* | KC | 2014/5/22 | - | - |
| CDApel9 | KP719783 | *Alopias pelagicus* | KC | 2014/5/22 | - | - |
| CDApel10 | KP719784 | *Alopias pelagicus* | KC | 2014/5/22 | - | - |
| CDApel11 | KP719785 | *Alopias pelagicus* | KC | 2014/5/22 | - | - |
| CDApel12 | KP719786 | *Alopias pelagicus* | KC | 2014/5/22 | - | - |
| CDAsup1 | KP719787 | *Alopias superciliosus* | KC | 2014/3/12 | - | - |

Continued

| Code | Accession # | Species | Location | Date | Weight (g) | Shape |
| --- | --- | --- | --- | --- | --- | --- |
| CDAsup2 | KP719788 | *Alopias superciliosus* | KC | 2014/5/3 | - | - |
| CDAsup3 | KP719789 | *Alopias superciliosus* | KC | 2014/5/22 | - | - |
| CDAsup4 | KP719790 | *Alopias superciliosus* | KC | 2014/5/22 | - | - |
| CDCcal1 | KP719791 | *Callorhinchus callorynchus* | KC | 2013/12/9 | - | - |
| CDCcal2 | KP719792 | *Callorhinchus callorynchus* | KC | 2013/12/9 | - | - |
| CDCcal3 | KP719793 | *Callorhinchus callorynchus* | KC | 2013/12/9 | - | - |
| CDCcal4 | KP719794 | *Callorhinchus callorynchus* | KC | 2013/12/9 | - | - |
| CDCcal5 | KP719795 | *Callorhinchus callorynchus* | KC | 2013/12/9 | - | - |
| CDCcal6 | KP719796 | *Callorhinchus callorynchus* | KC | 2013/12/9 | - | - |
| CDCcal7 | KP719797 | *Callorhinchus callorynchus* | KC | 2013/12/9 | - | - |
| CDCcal8 | KP719798 | *Callorhinchus callorynchus* | KC | 2013/12/9 | - | - |
| CDCcal9 | KP719799 | *Callorhinchus callorynchus* | KC | 2013/12/9 | - | - |
| CDCcal10 | KP719800 | *Callorhinchus callorynchus* | KC | 2013/12/9 | - | - |
| CDCcal11 | KP719801 | *Callorhinchus callorynchus* | KC | 2013/12/9 | - | - |
| CDCcal12 | KP719802 | *Callorhinchus callorynchus* | KC | 2013/12/9 | - | - |
| CDCcal13 | KP719803 | *Callorhinchus callorynchus* | KC | 2013/12/9 | - | - |
| CDCcal14 | KP719804 | *Callorhinchus callorynchus* | KC | 2013/12/9 | - | - |
| CDCcal15 | KP719805 | *Callorhinchus callorynchus* | KC | 2013/12/9 | - | - |
| CDCcal16 | KP719806 | *Callorhinchus callorynchus* | KC | 2013/12/9 | - | - |
| CDCmil1 | KP719807 | *Callorhinchus milii* | KC | 2013/12/9 | - | - |
| CDCmil2 | KP719808 | *Callorhinchus milii* | KC | 2013/12/9 | - | - |
| CDCmil3 | KP719809 | *Callorhinchus milii* | KC | 2013/12/9 | - | - |
| CDCmil4 | KP719810 | *Callorhinchus milii* | KC | 2013/12/9 | - | - |
| CDCacr1 | KP719811 | *Carcharhinus acronotus* | KC | 2013/12/9 | - | - |
| CDCfal1 | KP719812 | *Carcharhinus falciformis* | KC | 2014/3/24 | - | - |
| CDCfal2 | KP719813 | *Carcharhinus falciformis* | KC | 2014/3/24 | - | - |
| CDCfal3 | KP719814 | *Carcharhinus falciformis* | KC | 2014/3/24 | - | - |
| CDCfal4 | KP719815 | *Carcharhinus falciformis* | KC | 2014/3/24 | - | - |
| CDCfal5 | KP719816 | *Carcharhinus falciformis* | KC | 2014/3/24 | - | - |
| CDCfal6 | KP719817 | *Carcharhinus falciformis* | KC | 2014/3/24 | - | - |
| CDCfal7 | KP719818 | *Carcharhinus falciformis* | KC | 2014/3/12 | - | - |
| CDCfal8 | KP719819 | *Carcharhinus falciformis* | KC | 2014/3/12 | - | - |
| CDCfal9 | KP719820 | *Carcharhinus falciformis* | KC | 2013/12/4 | - | - |
| CDClon1 | KP719821 | *Carcharhinus longimanus* | KC | 2014/3/24 | - | - |
| CDClon2 | KP719822 | *Carcharhinus longimanus* | KC | 2014/3/24 | - | - |
| CDClon3 | KP719823 | *Carcharhinus longimanus* | KC | 2014/3/24 | - | - |
| CDClon4 | KP719824 | *Carcharhinus longimanus* | KC | 2014/5/8 | - | - |
| CDClon5 | KP719825 | *Carcharhinus longimanus* | KC | 2014/5/8 | - | - |
| CDClon6 | KP719826 | *Carcharhinus longimanus* | KC | 2014/5/15 | - | - |
| CDClon7 | KP719827 | *Carcharhinus longimanus* | KC | 2014/5/15 | - | - |

Continued

| Code | Accession # | Species | Location | Date | Weight (g) | Shape |
| --- | --- | --- | --- | --- | --- | --- |
| CDClon8 | KP719828 | *Carcharhinus longimanus* | KC | 2014/5/15 | - | - |
| CDClon9 | KP719829 | *Carcharhinus longimanus* | KC | 2014/3/12 | - | - |
| CDCsor1 | KP719830 | *Carcharhinus sorrah* | KC | 2013/12/9 | - | - |
| CDIoxy1 | KP719831 | *Isurus oxyrinchus* | KC | 2014/3/24 | - | - |
| CDIoxy2 | KP719832 | *Isurus oxyrinchus* | KC | 2013/12/9 | - | - |
| CDIoxy3 | KP719833 | *Isurus oxyrinchus* | KC | 2013/12/9 | - | - |
| CDIoxy4 | KP719834 | *Isurus oxyrinchus* | KC | 2013/12/9 | - | - |
| CDIoxy5 | KP719835 | *Isurus oxyrinchus* | KC | 2013/12/9 | - | - |
| CDIoxy6 | KP719836 | *Isurus oxyrinchus* | KC | 2013/12/9 | - | - |
| CDIoxy7 | KP719837 | *Isurus oxyrinchus* | KC | 2013/12/9 | - | - |
| CDIpau1 | KP719838 | *Isurus paucus* | KC | 2014/3/24 | - | - |
| CDIpau2 | KP719839 | *Isurus paucus* | KC | 2014/5/3 | - | - |
| CDMlun1 | KP719840 | *Mustelus lunulatus* | KC | 2013/12/9 | - | - |
| CDMpun1 | KP719841 | *Mustelus punctulatus* | KC | 2013/12/9 | - | - |
| CDPgla1 | KP719842 | *Prionace glauca* | KC | 2014/3/24 | - | - |
| CDPgla2 | KP719843 | *Prionace glauca* | KC | 2014/3/24 | - | - |
| CDPgla3 | KP719844 | *Prionace glauca* | KC | 2014/3/24 | - | - |
| CDPgla4 | KP719845 | *Prionace glauca* | KC | 2014/3/24 | - | - |
| CDPgla5 | KP719846 | *Prionace glauca* | KC | 2014/3/24 | - | - |
| CDPgla6 | KP719847 | *Prionace glauca* | KC | 2014/3/24 | - | - |
| CDPgla7 | KP719848 | *Prionace glauca* | KC | 2014/3/24 | - | - |
| CDPgla8 | KP719849 | *Prionace glauca* | KC | 2014/3/24 | - | - |
| CDPgla9 | KP719850 | *Prionace glauca* | KC | 2014/3/24 | - | - |
| CDPgla10 | KP719851 | *Prionace glauca* | KC | 2014/3/24 | - | - |
| CDPgla11 | KP719852 | *Prionace glauca* | KC | 2014/3/24 | - | - |
| CDPgla12 | KP719853 | *Prionace glauca* | KC | 2014/7/11 | - | - |
| CDPgla13 | KP719854 | *Prionace glauca* | KC | 2014/7/11 | - | - |
| CDPgla14 | KP719855 | *Prionace glauca* | KC | 2014/7/11 | - | - |
| CDPgla15 | KP719856 | *Prionace glauca* | KC | 2014/5/3 | - | - |
| CDPgla16 | KP719857 | *Prionace glauca* | KC | 2014/5/3 | - | - |
| CDPgla17 | KP719858 | *Prionace glauca* | KC | 2014/5/3 | - | - |
| CDPgla18 | KP719859 | *Prionace glauca* | KC | 2014/5/8 | - | - |
| CDPgla19 | KP719860 | *Prionace glauca* | KC | 2014/5/8 | - | - |
| CDPgla20 | KP719861 | *Prionace glauca* | KC | 2014/5/8 | - | - |
| CDPgla21 | KP719862 | *Prionace glauca* | KC | 2014/5/8 | - | - |
| CDPgla22 | KP719863 | *Prionace glauca* | KC | 2014/5/8 | - | - |
| CDPgla23 | KP719864 | *Prionace glauca* | KC | 2014/5/8 | - | - |
| CDPgla24 | KP719865 | *Prionace glauca* | KC | 2014/7/11 | - | - |
| CDPgla25 | KP719866 | *Prionace glauca* | KC | 2014/7/11 | - | - |
| CDPgla26 | KP719867 | *Prionace glauca* | KC | 2014/7/11 | - | - |

Continued

| Code | Accession # | Species | Location | Date | Weight (g) | Shape |
| --- | --- | --- | --- | --- | --- | --- |
| CDPgla27 | KP719868 | *Prionace glauca* | KC | 2014/3/12 | - | - |
| CDPgla28 | KP719869 | *Prionace glauca* | KC | 2014/3/12 | - | - |
| CDPgla29 | KP719870 | *Prionace glauca* | KC | 2014/3/12 | - | - |
| CDPgla30 | KP719871 | *Prionace glauca* | KC | 2014/3/12 | - | - |
| CDPgla31 | KP719872 | *Prionace glauca* | KC | 2014/3/12 | - | - |
| CDPgla32 | KP719873 | *Prionace glauca* | KC | 2014/3/12 | - | - |
| CDPgla33 | KP719874 | *Prionace glauca* | KC | 2014/3/12 | - | - |
| CDPgla34 | KP719875 | *Prionace glauca* | KC | 2014/3/12 | - | - |
| CDPgla35 | KP719876 | *Prionace glauca* | KC | 2014/3/12 | - | - |
| CDPgla36 | KP719877 | *Prionace glauca* | KC | 2014/3/12 | - | - |
| CDPgla37 | KP719878 | *Prionace glauca* | KC | 2014/3/24 | - | - |
| CDPgla38 | KP719879 | *Prionace glauca* | KC | 2013/12/4 | - | - |
| CDPgla39 | KP719880 | *Prionace glauca* | KC | 2013/12/4 | - | - |
| CDPgla40 | KP719881 | *Prionace glauca* | KC | 2013/12/9 | - | - |
| CDPgla41 | KP719882 | *Prionace glauca* | KC | 2013/12/9 | - | - |
| CDRacu1 | KP719883 | *Rhizoprionodon acutus* | KC | 2013/12/9 | - | - |
| CDRacu2 | KP719884 | *Rhizoprionodon acutus* | KC | 2013/12/9 | - | - |
| CDRacu3 | KP719885 | *Rhizoprionodon acutus* | KC | 2013/12/9 | - | - |
| CDRacu4 | KP719886 | *Rhizoprionodon acutus* | KC | 2013/12/9 | - | - |
| CDRacu5 | KP719887 | *Rhizoprionodon acutus* | KC | 2013/12/9 | - | - |
